# BLM regulates MALT1-driven NF-κB signalling and is targetable in B-cell malignancies

**DOI:** 10.1038/s41419-026-08846-3

**Published:** 2026-05-20

**Authors:** Ritu Agrawal, Supratim Ghosh, Nitin Kumar, Chetana Mukherjee, Vandana Sharma, Rimpy Arun, Riya Deb, Sumanta Sarkar, Savita, Dilip Kumar, Satyajit Rath, Arindam Maitra, Sagar Sengupta

**Affiliations:** 1 Biotechnology Research and Innovation Council—National Institute of Immunology (BRIC- NII), Aruna Asaf Ali Marg, New Delhi, 110067 India; 2 Biotechnology Research and Innovation Council—National Institute of Biomedical Genomics (BRIC-NIBMG), Kalyani, 741251 West Bengal India; 3 Indian Institute of Science Education and Research, Pune, 411008 Maharashtra India

**Keywords:** Haematological cancer, Immunology

## Abstract

Loss of BLM helicase leads to Bloom Syndrome, characterized by genomic instability, cancer predisposition and immunodeficiency. We now show that BLM is essential for the proliferation of cycling B cells and sustains B-cell development by maintaining NF-κB signalling. Hence, in the absence of BLM, the NF-κB pathway is impaired as visualized by the lack of the nuclear translocation of RelA. This action of BLM is due to its binding to the MALT1 promoter (a key positive regulator of NF-κB signalling) and activating its transcription. Reintroduction of MALT1 and constitutively active IKKβ rescues B-cell development in BLM-deficient bone marrow and spleen cells of BLM knockout mice. This indicates that downregulation of MALT1 in BLM-deficient cells is the primary cause of deregulated NF-κB signalling and impaired B-cell development. Interestingly, the pro-proliferative role of BLM can be exploited in the treatment of B-cell malignancies. Here, we demonstrate that depletion of BLM (phenocopied by the inhibition of MALT1) suppresses the progression of lymphoma and leukaemia by inhibiting MALT1-dependent NF-κB signalling and sensitizing malignant B cells to chemotherapy. Together, our findings establish the BLM-MALT1-NF-κB axis as a critical regulator of B-cell development and demonstrate its therapeutic potential in B-cell malignancies. Hence, both upregulation and downregulation of BLM contribute to oncogenesis, underscoring the need to maintain its expression within a tightly controlled threshold.

## Introduction

B cells undergo programmed cycles of DNA damage and repair as a fundamental part of their development and function. These controlled genomic alterations, including V(D)J recombination and class switch recombination (CSR), are essential for generating a diverse and adaptable antibody repertoire necessary for robust immune responses [1]. Both processes depend on precise and tightly regulated DNA proliferation and repair machinery [2, 3]. Disruptions in these pathways can lead to immunodeficiency and predispose individuals to malignancies, such as leukaemia and lymphoma. One such example is seen in individuals afflicted with Bloom syndrome (BS), a rare autosomal recessive disorder caused by biallelic mutations in the BLM gene, which encodes a RecQ-like helicase crucial for genome stability [4]. BLM helicase plays dual roles in homologous recombination (HR) and non-homologous end joining (NHEJ), exerting both pro- and anti-recombinogenic activities to prevent excessive or aberrant recombination events [5–7]. It is to be noted that elevated BLM expression can contribute to cancer progression, myeloma formation, and therapeutic resistance [8–10]. Nevertheless, both deficiency or mutated or overexpressed BLM increase susceptibility to carcinogenesis [8, 11], indicating that maintaining optimal BLM levels is crucial for physiological functions.

In mouse models, loss of BLM in B and T lymphocyte lineages results in impaired lymphocyte development, proliferation, and function [12, 13]. B cell-specific BLM deletion reduces mature B cell populations (pre-B cells), while early progenitors remain intact [13]. Immunodeficiency is also very common in BS patients [14] who have low levels of IgM, IgA, and IgG [15–17]. The molecular basis for the B cell developmental block in BLM-deficient cells remains unclear. Key regulators of B cell development include recombination, DNA damage repair and proliferation signalling [18, 19]. The NF-κB pathway is the major pro-survival signalling pathway that plays a key role in B cell proliferation, differentiation, and antigen response [20, 21]. The NF-κB pathway is tightly regulated by the CBM (CARD11–BCL10–MALT1) complex, which integrates antigen receptor–derived signals to drive transcription of survival and proliferation genes. MALT1 functions as a dual regulator: (a) as a scaffold protein, it stabilizes the CBM complex and facilitates downstream signalling to IKK and NF-κB; (b) as a protease, its paracaspase activity cleaves negative regulators of NF-κB [22, 23].

To dissect the role of BLM in lymphopoiesis, we generated B-cell–specific BLM conditional knockout mice. Integrated scRNA-seq and functional assays revealed that BLM is indispensable for sustaining NF-κB signalling. Further, it was revealed that BLM functions as an upstream transcriptional activator of MALT1, a procaspase that acts as a critical driver of the NF-κB pathway. Reintroduction of either MALT1 or BLM or constitutively active IKKβ into BLM-deficient mice rescued B-cell development, indicating that in the absence of BLM, MALT1 downregulation leads to impairment in NF-kB signalling and B-cell development. Interestingly, this role of BLM can be utilised to treat B-cell malignancies. We demonstrate that in leukaemic and lymphoma cells, ablation of BLM results in the dampening of the NF-κB-MALT1 axis and the simultaneous accumulation of damaged DNA. Thus, treating these cancer cells lacking BLM with frontline chemotherapeutic drugs (like mitoxantrone or daunorubicin) enhances their efficacy by increasing apoptosis. Thus, while elucidating the role of BLM in B-cell development, we identified and provided evidence that BLM itself can be a viable target for future drug development for the treatment of B-cell malignancies.

## Material and method

### Reagents

All antibodies used are listed in Table S1. All recombinants used have been described in Table S2. All reagents (including chemicals, oligonucleotides, animals, deposited data and other materials) used are described in Table S3. All primers are described in Table S4.

### Mice

All animal studies were carried out at the National Institute of Immunology according to approved animal ethics protocols (IAEC/AQ/2015/137, IAEC/AQ/2016/1502, IAEC/AQ/2017/150, IAEC/AQ/2019/182). All mice were housed in specific pathogen-free environments. Mice were randomly assigned to experimental groups to ensure unbiased group allocation. Sex was not a determinant. BLM B cell-specific knockout has been generated by crossing the *Blm tm4Ches*/J/J and transgenic CD79-cre mice, which generates F1 hybrids. The F1 generation was intercrossed to obtain BLM wild-type mice (WT), BLM hetero-knockout mice (HKO), and BLM knockout mice (KO). The CD79a Cre allele in all BLM genotypes was maintained in a heterozygous condition.

Xenograft studies were carried out in NSG mice with shControl Raji and shBLM Raji cells. 3 × 10^6^ cells were mixed with matrigel (1:1 ratio) and then injected subcutaneously. In all cases, tumour volume was measured at the indicated days post-injection using the formula: Tumor volume = 1⁄2 (length × width^2^).

Engraftment experiments were performed using NSG mice injected with either shControl or shBLM in RCH-ACV cells. 6 × 10^6^ cells were administered via tail vein injection. Disease progression was assessed periodically by collecting peripheral blood through retro-orbital bleeds, followed by flow cytometric analysis using an anti-human CD19 antibody to detect human leukemic cells. On day 30, mice were euthanized, and leukemic cells were isolated from bone marrow and spleen for further flow cytometry analysis.

### Cell lines

Raji cells, RCH-ACV, Daudi and NAMLWA were grown in RPMI-1640 media. HEK293T, HEK293T TLCV2 and HEK293T TLCV2 BLM sgRNA cells were grown in DMEM media. In all cases, the media were supplemented with 10% fetal bovine serum, L-glutamine or sodium pyruvate, and Penicillin, Streptomycin and Amphotericin B. Cells were maintained at 37 °C and 5% CO_2_. All stable lines (Raji and RCH-ACV expressing either shControl or shBLM or shControl expressing MALT1 or shBLM-expressing MALT1) were additionally supplemented with 1X non-essential-amino acids, 1X Vitamin solution. shControl and shBLM selected in 2 µg/ml puromycin and post-selection grown in 1 µg/ml puromycin. shControl expressing MALT1 and shBLM-expressing MALT1 were selected in 100 µg/ml hygromycin and post-selection grown in 50 µg/ml hygromycin. Cells were treated for 24 h with either 12.5μM ML216 (BLM helicase inhibitor) or 1 μm MI-2 (MALT1 inhibitor).

### Cell isolation

The bone marrow and spleen of 6–12-week-old mice were used to isolate cells. Bone marrow was isolated from the femurs and tibia, and the bones were flushed from one end with RPMI media without any supplement. Total cells were collected, washed with 1XPBS, and centrifuged at 2000 rpm for 3 min. The spleen was minced using frosted glass slides while immersed in 1XPBS, then centrifuged. RBC was lysed using a filter-sterilized 0.15 mM NH_4_Cl solution for 20 min. Cells were washed with 1XPBS.

### FACS sorting

Single-cell suspensions from the spleen and bone marrow post-RBC lysis were used for FACS analysis. For this purpose, the suspension was incubated with anti-CD45R/B220 antibody for 15 min. The cells were washed with 1XPBS and filtered through a 70 μm cell strainer. Cells were sorted using a multicolour BD Aria III. Sorted cells were collected in FACS buffer (1:1 ratio of undiluted FBS and 1XPBS).

### FACS analysis

Cells were processed similarly to FACS sorting, as described above. B cell immunophenotyping was performed using anti-B220, anti-CD43, anti-CD23 and anti-CD21/35 antibodies. The BD FACSVerse flow cytometer was used to acquire the data, while FlowJo software was used to analyze the flow cytometry data.

### Magnetic-activated Cell Sorting (MACS)

Total spleen cells after RBC lysis were incubated with 80 µl of CD45R (B220) microbeads for 15 min in an end-to-end rocker at room temperature. The LS columns were placed on a magnetic stand and equilibrated with 5 mL MACS buffer (10% FBS, 2 mM EDTA, 1X PBS). The cell suspension was added to the column and washed twice with 5 ml MACS buffer. B220⁺ cells were eluted by removing the column from the magnetic field and flushing with 5 ml of MACS buffer.

### Genotyping

DNA was isolated from the tails using the Quick DNA isolation protocol of the Jackson Laboratory (https://www.jax.org/jax-mice-and-services/customer-support/technical-support/genotyping-resources/dna-isolation-protocols). PCR was set up to amplify the region surrounding Cre (exons 2 and 3) and BLM (exon 7), with the primers as mentioned [24]. The products were resolved on 3% agarose gels. To further confirm the deletions in the B cells, DNA was isolated from FACS-sorted B220+ spleen B cells using the PicoPure® DNA extraction kit as per the manufacturer’s guidelines. 10–50 ng of DNA was used for Long Amp PCR to confirm the BLM exon 7 deletion, as mentioned in [24].

### Southern blot

Genomic DNA was extracted from FACS- or MACS-sorted B220⁺ B cells isolated from the spleens of WT, HKO, and KO mice. A total of 15 µg of genomic DNA was digested overnight with HindIII, following the procedure described in [24].

### ChIP and ChIP seq

Total spleen cells after RBC lysis were diluted with 5 ml and 15 ml of 1XPBS, respectively. Mice spleen cells or human cells (Raji or RCH-ACV) were fixed by adding 27 μl of 37% formaldehyde per ml of 1XPBS-containing cells, incubated for 8 min at room temperature, and quenched with 115 μl of 0.125 M glycine per ml of 1XPBS-containing cells for 5 min. After washing with 1XPBS, blocking was performed for 1 min using 200 μl of 5% BSA per ml of 1XPBS-containing cells. Cells were stained with anti-B220 antibody conjugated to PE-Cy7. B220+ cells were sorted and washed with 1XPBS. The pellet was resuspended in 25 μl (per 70,000 cells) of nuclear lysis buffer (10% SDS, 0.5 M EDTA, 1 M Tris-HCl, pH 8.0 supplemented with 1X PIC) and incubated on ice for 1 h. ChIP dilution buffer (1% Triton X-100, 2 mM EDTA, 20 mM Tris–HCl pH 8.1, 150 mM NaCl supplemented with 1X Protease Inhibitor Cocktail) was added (75 µl per 70,000 cells), and the samples were sonicated at 4 °C using a Bioruptor Plus (30 s pulses on and off, 40 cycles) to shear the chromatin so that approximately 400 bp fragments were obtained. After checking the size of the sheared chromatin in a 1% agarose gel, the samples were centrifuged at 13,000 rpm for 10 min at 4 °C, and the supernatant containing the sheared chromatin was collected. Sheared chromatin was diluted 1:10 using the ChIP dilution buffer containing 2XPIC. ChIP sequencing was performed using the True Micro ChIP-seq Kit with 1 μg of BLM antibody and 10 μl of Protein G beads, incubated overnight at 4°C in an end-to-end rotor. After the reaction, all steps were performed according to the manufacturer’s guidelines. Eluted ChIP DNA was quantified using Qubit.

### RT-qPCR and ChIP-qPCR

Total RNA was isolated from B220+ cells using the TRIzol LS Reagent. RNA concentrations were determined using Qubit. cDNA was generated using the Reverse Transcriptase Core Kit according to the manufacturer’s protocol. 100 ng of cDNA was used for RT-qPCR. The concentrations of input samples and ChIP DNA were determined by Qubit using a dsDNA HS assay kit. 1 ng of ChIP DNA was used for ChIP-qPCR with primers that amplify regions on MALT1 promoters. According to the manufacturer’s instructions, real-time RT-qPCR was carried out with the SYBR green RT-qPCR kit using the QuantStudio 3 Real-time PCR system. The relative level of gene expression was determined using the 2^(-ΔΔCt)^ method.

### ChIP-seq analysis

The analysis was carried out following the methodology described in a previous study [25], with the exception that read alignment was conducted using the mm39 genome reference (GRCm39, GCA_000001635.9, June 2020). Briefly, the quality of the raw reads was determined using FastQC (http://www.bioinformatics.babraham.ac.uk/projects/fastqc/), followed by removal of adapters using Trim Galore (https://www.bioinformatics.babraham.ac.uk/projects/trim_galore/). Bases with a high Phred score (more than 30) were aligned to the mm39 genome reference using BWA [26]. Peaks were detected using MACS2 and annotated using the ChIPseeker R package.

### Western blot

The B220 + FACS-sorted spleen cell lysates were prepared using RIPA buffer (1 mM Tris HCl, pH 7.8, 150 mM NaCl, 2% Triton X-100, 1% (w/v) sodium deoxycholate, and 0.1% (w/v) SDS), supplemented with 1XPIC for lysate preparation. Lysate protein concentrations were assessed using Bradford reagent, and 20 µg of lysate was used for immunoblotting with the indicated antibodies. Subsequently, 6X SDS-PAGE loading buffer (composition: 375 mM Tris HCl pH 6.8, 6% SDS, 48% glycerol, 9% β-mercaptoethanol and bromophenol blue) was added to the lysates, boiled for 10 min, and SDS-PAGE followed by western blotting was carried out. All original and uncropped western blots are included in the Supplemental Material.

### Nuclear extraction

Cytoplasmic and nuclear fractions were isolated using NE-PER™ extraction reagents as per the manufacturer’s instructions. 1 × 10^6^ cells were lysed in CERI and CERII reagents on ice to obtain cytoplasmic fractions, while intact nuclei pellets were suspended in NER reagents to release nuclear contents.

### ELISA assay

RelA (p65) and phospho RelA (pS536) levels were quantified through an NF-kB p65 Kit according to the manufacturer’s guidelines. To assess total p65, 10 μg of B220+ nuclear lysate was used. To quantify phospho-RelA (pS534), 40 μg of B220+ bone marrow spleen whole-cell lysate was used. Lysates were diluted with cell extraction buffer, incubated for 1 h at room temperature with 40 μl of the respective antibody cocktail in antibody-coated wells. Post-incubation, 100 μl of the substrate was added, and readings were taken using an ELISA plate reader at 450 nm after adding the stop solution.

### Serum cytokine and interleukin levels

Blood was collected via retro-orbital bleeding and allowed to clot at room temperature for 30 min. Samples were then centrifuged at 1000 × *g* for 10 min to separate the serum. The collected serum was subjected to a second centrifugation at 1000 × *g* to remove residual platelets and cellular debris. Cleared serum samples were aliquoted and stored at –80 °C until analysis. Prior to analysis, samples were kept at 4 °C overnight. Cytokine and interleukin levels were quantified using the ProcartaPlex™ Mouse Immune Response Panel 64-Plex (Thermo Fisher Scientific), following the manufacturer’s protocol.

### MTT assay

50,000 cells were seeded in a 96-well plate, followed by treatment with Mitoxantrone (0.1 μM to 3 μM) or Daunorubicin (0.1 μM to 2 μM) for 48 h. Post treatment, 10μl of 5 mg/ml MTT was added to each well, incubated for 4 h at 37 °C and solubilized with 100 μl solubilizing buffer. Readings were taken at 570 nm on a microplate reader, and then the percentage cell viability was calculated.

### Immunofluorescence

B220+ cells were seeded on a poly-L-lysine-coated coverslip, flame sterilized with 100% ethanol in a 24-well plate and centrifuged at 1200 rpm for 5 min. Cells were fixed with 4% PFA for 5 min at room temperature. Post-fixation cells were washed twice with 1X PBS for 8 min, and cells were permeabilized with 1X PBS containing 0.1% Triton-X-100 for 5 min. Subsequently, the cells were washed twice for 8 min with 1XPBS and blocked with 10% BSA in 1XPBS for 1 h. Post-incubation, cells were washed, incubated with the respective antibodies for 1 h at room temperature, followed by washing, and then again incubated with their respective secondary antibodies for 1 h at room temperature. The coverslips were mounted on frosted glass slides with a mounting medium containing DAPI. Cells were visualized and imaged at 63×/1.4 magnification in a Zeiss LSM980 Meta confocal microscope. Laser lines used for imaging were 405/561/647 nm.

### Cre-inducible retroviral vector construction

A retroviral vector, MSCV-LoxP-DSRed2N1-LoxP-DEST-IRES-Puro-T2A-Thy1.1, was constructed for Cre recombinase-inducible overexpression of target genes, based on the strategy described in [27] with specific modifications. The vector backbone was derived from pMSCV-loxP-dsRed-loxP-eGFP-Puro-WPRE. To enable gateway-based cloning, a DEST cassette along with an internal ribosome entry site (IRES) was amplified from the plenti-CAG-gate-FLAG-IRES-GFP plasmid. This fragment was inserted into the pMSCV-loxP-dsRed- loxP-eGFP-Puro-WPRE vector between the loxP-flanked dsRed and puromycin resistance cassette using ApaI and HindIII digestion followed by In-Fusion Cloning, resulting in the intermediate pMSCV-loxP-dsRed-loxP-DEST-IRES-Puro-WPRE construct. To enable co-expression of a surface marker, a synthetic sequence encoding the F2A self-cleaving peptide in-frame with Thy1.1 was designed and synthesized by IDT. This F2A-Thy1.1 fragment was inserted downstream of the puromycin resistance gene and upstream of the WPRE element via In-Fusion Cloning into the NsiI-digested pMSCV-loxP-dsRed-loxP-DEST-IRES-Puro-WPRE vector, yielding the final construct: MSCV-LoxP-DSRed2N1-LoxP-DEST-IRES-Puro-T2A- Thy1.1.

### Retrovirus generation

HEK293T cells were seeded in a 10-cm plate for virus production. At 70–80% confluency, co-transfection of retroviral expression plasmid, namely MSCV-TraSTOP-GFP-IRES-Puro-T2A-Thy1.1, MSCV-TraSTOP-murine-BLM-IRES-Puro-T2A-Thy1.1, MSCV-TraSTOP-murine MALT1-IRES-Puro-T2A-Thy1.1, retro-gfp-puro vector, retro-gfpIkkb-puro vector (murine), retro-gfpIkkb (S177E, S181E)-puro vector (murine) and pBABE FLAG-IKK2-EE puro (human) together with pCL-Eco (retroviral packaging and envelop plasmids) in a 1:1 ratio was carried out using Lipofectamine 3000 (in a 1:2 ratio). Subsequent processing steps were carried out following the lentiviral production protocol described in [9].

### Stable cell line generation

To generate Raji and RCH-ACV expressing either shControl or shBLM using pLKO.1 Puro shcontrol or pLKO.1 Puro shBLM were used. To express MALT1, wild-type BLM, BLM helicase dead (K695A), pLVX-IRES-hygro-Flag MALT1, pLVX-IRES-hygro-Flag BLM, pLVX-IRES-hygro-Flag BLM K695A were used as described in [25].

### Transfections

Plasmid transfections were carried out by Lipofectamine 2000 in HEK293T cells, and by X-tremeGENE^TM^ HP in Raji cells according to the manufacturer’s protocol for 24–60 h. For luciferase assays, 0.5 µg of the luciferase reporter and 0.5 µg β-galactosidase mammalian overexpression plasmids were co-transfected.

### Human PBMC and CD19+ cells isolation

Human blood was collected at the National Institute of Biomedical Genomics according to approved human ethics protocols (NIMBG/2025/2/0071). Written informed consent was obtained from all participants prior to their inclusion in the study. Sex was not a determinant. Peripheral blood mononuclear cells (PBMCs) were isolated from whole blood using density-gradient centrifugation with HiSep™ LSM 1077. Briefly, 10 ml of whole blood was diluted 1:1 with sterile 1X PBS. 20 ml diluted blood was carefully layered over 7 ml of HiSep™ LSM 1077 in a 50 ml centrifuge tube and centrifuged at 400 × *g* for 30 min at 25 °C without brake. The PBMC layer at the plasma–LSM interface was collected and washed with 1X PBS by centrifugation at 400 × *g* for 5 min at 25 °C. The cell pellet was resuspended in 5 ml RBC lysis buffer (0.15 mM ^NH^4^Cl^) and incubated for 5 min at room temperature to remove residual erythrocytes, followed by the addition of 20 ml 1X PBS and centrifugation at 400 × *g* for 5 min at 25 °C. To remove platelets, cells were resuspended in 20 ml 1X PBS and centrifuged at 100 × *g* for 10 min at 25 °C. Finally, PBMCs were resuspended in freezing media and stored at −80 °C. PBMCs were thawed in complete RPMI medium (10% FBS, 1% L-glutamine, 1% vitamin solution, 1% non-essential amino acids, and 50 μM β-mercaptoethanol), centrifuged, and washed with 1× PBS at 25 °C. Cells were resuspended in 1 ml 1X PBS containing 0.1% BSA and 2 mM EDTA (resuspension buffer). Anti-CD19 Dynabeads (20 μl) were added and incubated for 15 min at room temperature on an end-to-end rotor. The tube was placed on a magnetic stand and washed three times with the same buffer. Bead-bound cells were then resuspended in 100 μl resuspension buffer, and 10 μl DETACHaBEAD CD19 was added and incubated for 45 min at room temperature. After magnetic separation, the supernatant containing detached cells was collected, washed with 10 ml complete RPMI medium, and seeded.

### Apoptosis assay

Apoptosis was assessed using the Membrane Permeability/Dead Cell Apoptosis Kit with PO-PRO™ -1, and 7-Aminoactinomycin D (7-AAD) was used for Flow Cytometry to detect apoptosis. Briefly, cells were harvested, washed in cold 1X PBS, and the cell density was adjusted to 1 × 10^6^ cells/ml in PBS. 2.5 µL PO-PRO™ −1 and 1 µL 7-AAD stock solution were added to each 1 ml of cell suspension, and the cells were incubated on ice for 30 min. Cells were then washed with 1× PBS and immediately analyzed by flow cytometry using violet and 488 nm excitation.

### γH2AX staining

Cells were harvested, washed with 1× PBS, and fixed with 4% paraformaldehyde (PFA) for 10 min at room temperature on an end-to-end rotor. Cells were then washed with 1× PBS and permeabilized with 0.2% Triton X-100 in PBS for 10 min at 25 °C. After washing, cells were incubated with PE-conjugated γH2AX antibody (1:100) in 3% BSA for 1 h at room temperature. Following incubation, cells were washed with 1× PBS, resuspended in 100 μl PBS, and analyzed by flow cytometry.

### scRNA sample processing

The mouse bone marrow (Fraction A-C’ and D) and spleen (B220 + ) cells were sorted and cryopreserved in Cryostor CS10. Cells were revived by thawing in a water bath at 37 °C for 30 s, and warm RPMI containing 10% FBS and 1% L-glutamine was added and centrifuged at 300xg for 5 min. This process was repeated twice, after which cells were washed twice in either FACS buffer or MACS buffer at room temperature. Fc receptors were blocked by TruStain FcX™ PLUS anti-mouse CD16/32 (0.25 μg/10^6^ cells) in 50μl suspension by incubating on ice for 10 min. Cells were tagged with 0.25μg hashtag oligo (HTO) diluted in 50 μL buffer, followed by centrifugation at 14,000 × *g* at 4 °C for 10 min before adding to the cells to ensure the removal of protein aggregates. HTO solution was carefully pipetted from the top, added to 50 µl cell suspension, and incubated for 30 min at 4 °C. Cells were washed twice with 1 ml FACS/MACS buffer. Upon reaching a concentration of 1000–2000 cells/µl, equal volumes from samples having unique HTO antibody tagging were pooled to generate a single-cell suspension for scRNA-Seq.

### scRNA seq library preparation and sequencing

Using the single-cell suspension, the 5’-gene expression libraries were prepared as prescribed by the manufacturer [Chromium Single Cell 5’ Reagent Kits User Guide (v2 Chemistry Dual Index) with Feature Barcoding technology for Cell Surface Protein and Immune Receptor Mapping, CG000330 Rev G, 10X Genomics]. The master mix for reverse transcription, along with cell suspension, partitioning oil and single-cell VDJ 5ʹ Gel Bead, was added on Chromium Next GEM Chip K and loaded on a Chromium controller droplet-based microfluidics system for ~18 min for Gel Bead-in Emulsion (GEM) generation. The mRNAs captured inside GEM were converted into cDNA by reverse transcriptase. The cDNA was cleaned and amplified using 5’ feature barcode cDNA primers. The supernatant, during the amplified cDNA clean-up step, was stored for cell-surface antibody-tagged oligonucleotide library preparation. 50 ng DNA input was taken from amplified cDNA to generate 5’ scRNA-Seq libraries. The library preparation steps included fragmentation, end-repair, A tailing, adapter ligation and sample index PCR by adding dual index (i5 and i7) from Dual Index Plate TT Set A. 5 µl from the stored supernatant containing cell-surface HTO fragments were used for library preparation using sample index PCR by adding dual index (i5 and i7) from Dual Index Plate TN Set A. RNA library and HTO library were pooled in a ratio of 4:0.8 for sequencing. The sequencing run was performed in the NovaSeq6000 using paired-end sequencing chemistry of 26-10-10-90 (R1- i5-i7-R2) (numbers refer to the number of cycles allocated to each read).

### scRNA-seq analysis

Binary Base Call files (raw sequence intensity file) using the CellRanger (https://www.10xgenomics.com/support/software/cell-ranger/latest) software suite (10X Genomics) mkfastq pipeline were demultiplexed and converted into FASTQ files [28]. Two FASTQ files (gene expression (GEX), VDJ, and antibody (HTO) for each pooled sample were generated. The quality of the FASTQ files was evaluated using the FASTQC (https://github.com/s-andrews/FastQC). FASTQ files were further demultiplexed to generate individual sample files and count matrices by aligning them to the mouse reference genome GRCm39 (https://cf.10xgenomics.com/supp/cell-exp/refdata-gex-GRCm39-2024-A.tar.gz) using the CellRanger v7.1.0 multi-pipeline. Next, the GEX cell × gene matrix was processed using the R package Seurat v4.4.0 (https://github.com/satijalab/seurat) [29]. Cells expressing less than 200 genes or mitochondrial read content exceeding 20% were excluded. Clustering was performed using the Louvain algorithm, and clusters were visualized with Uniform Manifold Approximation and Projection (UMAP). Doublets were identified and removed using the R package DoubletFinder v2.0.3 (https://github.com/chris-mcginnis-ucsf/DoubletFinder) [30]. For tissue-specific analysis (bone marrow and spleen), samples were integrated using the R package Harmony v1.2.3 (https://github.com/immunogenomics/harmony) [31] and marker genes for each cell cluster were identified using the Wilcoxon rank-sum test. All other cells were excluded after annotation, retaining only B cells and their subtypes. The retained B cells were re-clustered for final downstream analyses. Cell cycle stages for B cells were scored using the Seurat CellCycleScoring method [29]. Differentially expressed genes were identified using the MAST test implemented in the Seurat FindMarkers function, applying multiple testing corrections (FDR BH < 0.05). The identified upregulated (FC  \(\ge \)1.5) and downregulated (FC \(\le \) 0.66) genes with a *p* value < 0.05 were analyzed for pathway enrichment using the R packages EnrichR (https://github.com/wjawaid/enrichR) [32] and ClusterProfiler (https://github.com/YuLab-SMU/clusterProfiler) [33]. Cell-state enrichment was assessed using the R package MiloR (https://github.com/MarioniLab/miloR), which performed differential abundance testing of cell neighbourhoods between pairs of genotypes under each condition. Fold changes and statistical significance were calculated, with neighbourhoods considered significant at a spatial FDR < 0.1 [34].

### Statistical analysis

Quantitative data are reported as mean ± standard deviation. Information regarding the number of samples analyzed for each experiment is provided in the figure legends. The *p* values are provided in the corresponding figures. Details of the statistical test used for each experiment are provided in Table S5.

## Results

### Loss of BLM impairs NF-κB signalling via pro-caspase, MALT1

Previous studies have shown that BLM deficiency in B lymphocytes reduces total B cell numbers and immunoglobulin isotypes, accompanied by maturation defects [13]. BLM is known to be a DNA damage repair protein [4], and its absence may lead to the accumulation of DNA damage in B cells. To explore the mechanism underlying BLM’s role in B cell development, we generated a conditional BLM knockout (KO) mouse model using the CD79a-Cre promoter, ensuring B cell-specific deletion of BLM throughout the entire B cell lineage. BLM gene dosage was confirmed through genomic DNA PCR (Figure S1A) and Southern blot analysis (Figure S1B). Consistent with previous findings [13], analysis of 6- to 18-week-old mice demonstrated a reduction in total B220⁺ B cells in both the bone marrow and spleen of BLM heterozygous knockout (HKO) and homozygous knockout (KO) mice compared to their wild-type (WT) mice counterparts (Figure S1C, S1D, S1G, S1H). While pro-B (Fraction A-C’) cell numbers remained unchanged across genotypes, pre-B cell (Fraction D) populations were significantly decreased in both HKO and KO mice, with the most pronounced reduction observed in KO mice (Figure S1E, S1F), indicating BLM is critical for the pro- to pre-B cell transition. Interestingly, this defect progressively appears to impact follicular B cells, with a marked reduction linked to BLM gene dosage. (Figure S1G, S1I).

**Fig. 1:**
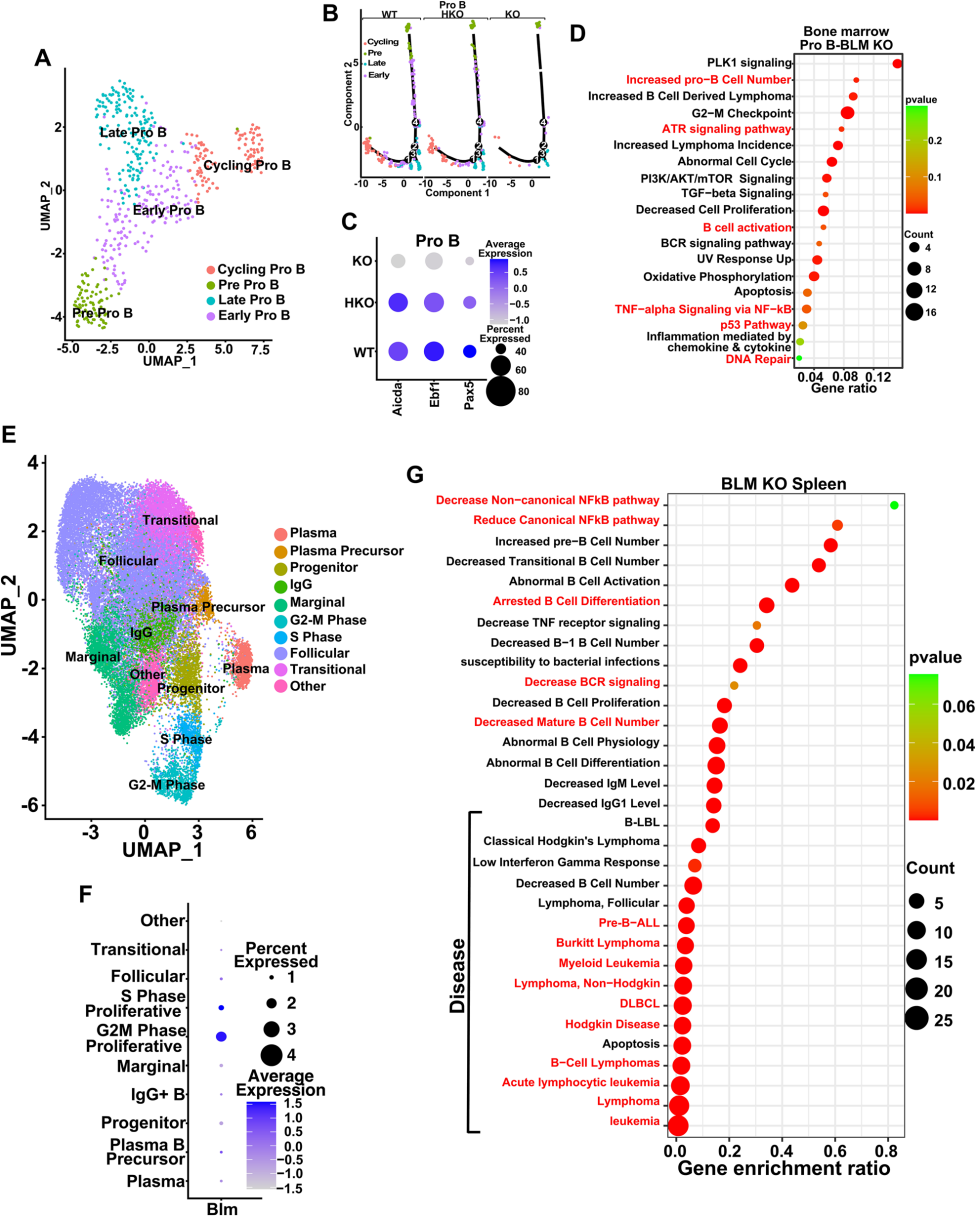
Role of BLM deficiency on pro-B and spleen-B cell development. **A** *Clustering of bone marrow pro-B cell subpopulations*. UMAP plots show different clusters representing pro-B cell states derived from scRNA-seq data from FACS-sorted bone marrow cells obtained from *n* = 3 mice per genotype (WT, HKO, and KO). For each genotype, samples were pooled using the B220⁺ hashtag labelling. FACS-sorted B220⁺CD43⁺ cells (Fractions A–C’), representing pro-B cell subpopulations. Each point denotes a single cell, colour-coded by Seurat clustering and annotated by cell states as inferred by the expression of canonical marker genes. **B** *Pseudotime mapping of pro-B cells highlights defective B cell development caused by BLM loss*. RNA velocity of pro-B was calculated in terms of pseudotime and the trajectory of cellular differentiation has been represented for the three genotypes. **C** *Reduced expression of B cell lineage transcription factors in BLM deficient pro-B cells*. For the three genotypes, dot plots have been presented to illustrate the expression of B cell lineage transcription factors (Ebf1, Pax5), CSR gene (Aicda) in pro-B cells. The dot size indicates the percentage of cells expressing each gene, while the intensity of the dot colour represents the average expression level. **D**, **G** *Pathway enrichment analysis indicates key B-cell mediated pathways are deregulated*. Pathway analysis was carried out using EnrichR in (**D**) pro-B and (**G**) spleen-B cells obtained from BLM KO mice. **E** *Clustering of splenic B-cell subpopulations*. UMAP representation of B cell subtypes using scRNA-seq data from B220 + MACS-sorted splenic B cells [*n* = 3 mice per genotype (WT, HKO, and KO)]. For each genotype, samples were pooled using the B220⁺ hashtag labelling. Cells are colour-coded by transcriptionally defined clusters. **F** *Expression of BLM is maximal in proliferative splenic B cells*. Dot plot illustrating the level of expression of BLM in different phases of the cell cycle and in the splenic B subpopulations of WT mice.

To delineate whether the impairment of B cell development upon BLM loss arises exclusively from a dysregulated DNA damage response or also involves additional mechanisms, we performed single-cell RNA sequencing (scRNA-seq) on FACS-purified pro-B (B220⁺CD43⁺), pre-B (B220⁺CD43⁻), and splenic B (B220⁺) cells isolated from BLM-WT, HKO, and KO mice. Pro-B cell subpopulations and spleen B cells were annotated based on their gene expression profiles (Figs. 1A, E and Figures. S2A, S2D, S2E). Pseudotime trajectory analysis revealed a marked disruption in the transition from pre-pro-B to early pro-B cells in BLM KO mice, resulting in a diminished pool of late pro-B and cycling pro-B cells (Fig. 1B and Figure. S2B). This reduction was accompanied by a notable impairment in the progression to the pre-B cell stage, thereby validating the FACS data (Figure. S1C, S1F). Importantly, BLM expression was highest in cycling pro-B cells (Figure S2C) and in G2-M and S phase splenic B cells (Fig. 1F), underscoring its essential role in promoting proliferation and maintaining genomic stability. Consistent with these findings, BLM KO pro-B cells showed significantly reduced expression of key early B cell development genes, including *Ebf1* and *Pax5*, indicating impaired differentiation and maturation (Fig. 1C). Pathway enrichment analysis further supported these observations, revealing deregulation of multiple processes critical to B cell development in both pro-B and splenic B cells. These included B cell activation, differentiation, cell cycle regulation, DNA damage signalling and DNA repair (ATR signalling, p53 pathway), NF-κB signalling and disease development (lymphoma, leukaemia) (Figs. 1D, G). Similar findings were evident in BLM KO pre-B cells (data not shown). Further analysis of scRNA-seq data from bone marrow pro-B cells also revealed reduced expression of Bcl2 and Bcl2l1, key NF-κB-regulated anti-apoptotic genes in BLM KO cells (Figure S3A), prompting us to test whether defective proliferation stems from impaired NF-κB signalling in BLM-deficient B cells.

**Fig. 2:**
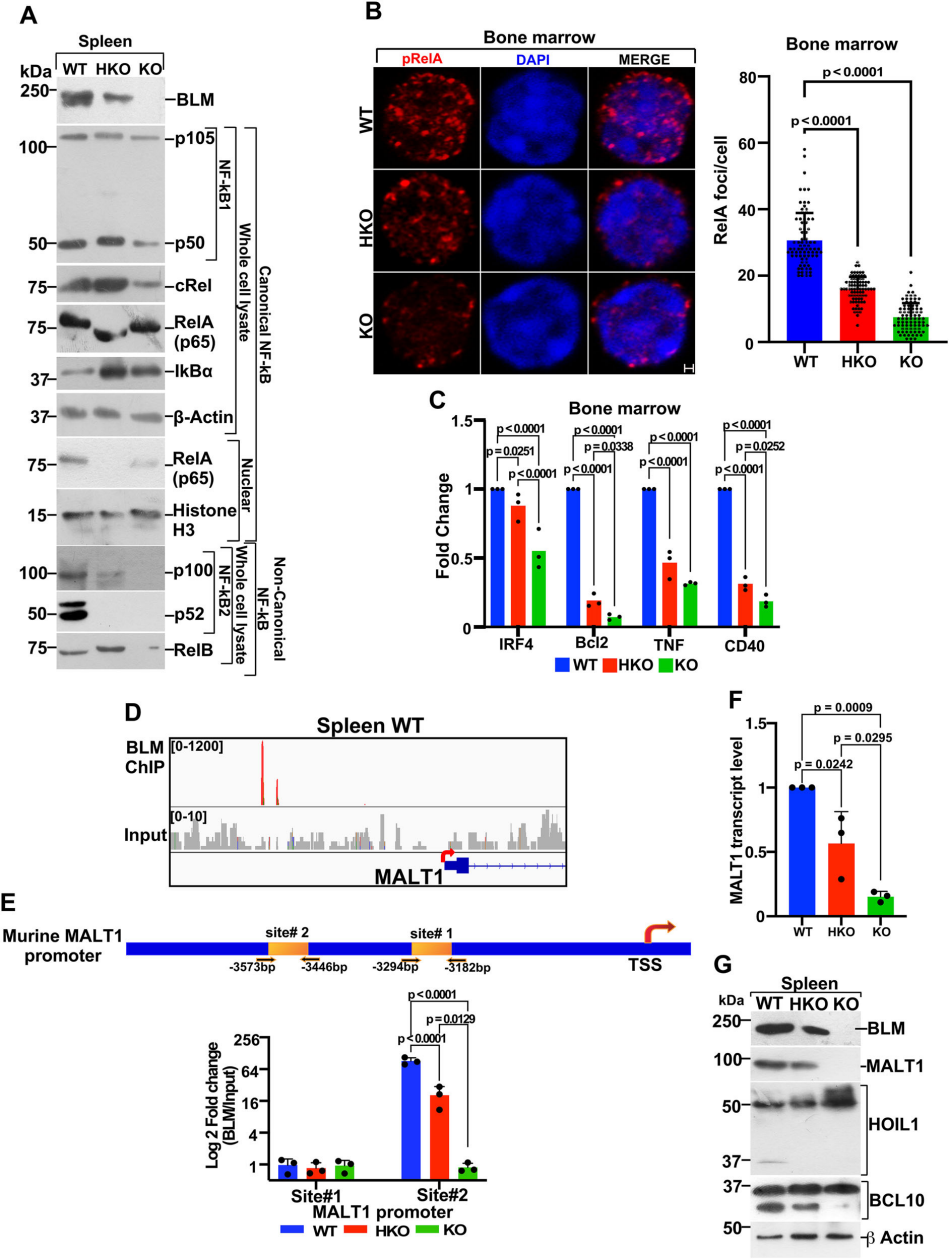
BLM deficiency leads to downregulation of the NF-κB signalling pathways. **A** *Both canonical and non-canonical NF-κB pathways were suppressed upon deletion of one or both BLM alleles*. Western blot analysis was performed using whole-cell extracts and nuclear lysates (as indicated) obtained from FACS-sorted spleen B220⁺ cells of WT, HKO, and KO mice. Blots were probed with the indicated antibodies. Representative data for one biological replicate. Three biological replicates were done (2 mice per genotype per biological replicate). **B** *Phospho-RelA levels were reduced in the absence of BLM in bone marrow*. (Left) Immunofluorescence was carried out on FACS-sorted B220⁺ cells from bone marrow using antibodies against phospho RelA (S536). DNA was counterstained with DAPI. Bar: 5 μm. (Right) Quantitation of immunofluorescence carried out in spleen cells for phospho RelA (S536) staining. Number of nuclei analysed in each case (*n* ≥ 80) from three biological replicates, *n* = 1 mouse per genotype per biological replicate. Mean ± SD. **C** *RelA targets are transcriptionally downregulated in BLM HKO and KO mice*. RNA was isolated from FACS-sorted B220 + B cells from bone marrow. RT-qPCR analysis of the indicated RelA targets was performed across all three genotypes. Data represent three biological replicates (1 mouse per genotype per biological replicate). Mean ± SD. **D** *BLM is recruited to the MALT1 promoter*. BLM ChIP-seq was performed on FACS-sorted B220⁺ cells from BLM WT mice. IGV browser tracks display prominent BLM enrichment peaks on the MALT1 promoter in splenic B cells, along with corresponding input signals. Only the region upstream of the MALT1 transcription start site (TSS) is shown. ChIP-seq data generated from *n* = 3 mice. **E** *BLM recruits to the MALT1 promoter at site #2*. (Top panel) Schematic representation of the mouse MALT1 promoter indicating the locations of the BLM ChIP primer binding sites. (Bottom panel) Extent of recruitment of BLM to site #1 (−3182 to −3294 w.r.t TSS), site #2 (−3446 to −3573 w.r.t TSS) and on the MALT1 promoter was determined by ChIP-qPCR using FACS-sorted spleen B220⁺ cells of BLM WT, HKO, and KO mice. Data represent three biological replicates (1 mouse per genotype per biological replicate). Mean ± SD. **F** *MALT1 transcript levels were reduced in a BLM dose-dependent manner*. RT-qPCR analysis of MALT1 mRNA levels was performed using FACS-sorted spleen B220⁺ cells of BLM WT, HKO and KO mice. Data represent three biological replicates (1 mouse per genotype per biological replicate). Mean ± SD. **G** *MALT1 protein levels and its activity were suppressed in the absence of BLM*. Whole-cell lysates were prepared from FACS-sorted spleen B220⁺ cells of BLM WT, HKO and KO mice. Immunoblotting was performed with the indicated antibodies. Data represent three biological replicates (2 mice per genotype per biological replicate).

For this purpose, we first assessed the levels and activities of the key components of both the canonical and non-canonical NF-κB pathways in FACS-sorted B220⁺ spleen cells. Western blot analysis showed a marked reduction in the processing of p105 (NF-κB1) to p50, along with a significant downregulation of c-Rel in cells from KO mice (Fig. 2A). Moreover, IκBα accumulated in both BLM HKO and KO cells. Given that IκBα degradation promotes RelA translocation to the nucleus [35], we next examined nuclear lysates to assess the extent of RelA activation. Although RelA levels in whole-cell extracts remained unchanged, we observed a substantial decrease in nuclear RelA levels in cells from both HKO and KO mice (Fig. 2A), indicating a lack of RelA activation in these cells. Immunofluorescence staining for phosphorylated RelA (pRelA-Ser536) showed markedly reduced pRelA in cells from both bone marrow and spleen of HKO and KO mice (Fig. 2B, Figure. S3B). Furthermore, ELISA for pRelA-Ser536 using whole-cell lysates from FACS-sorted B220⁺ spleen cells revealed a decline in pRelA levels in HKO and KO (Figure S3C, S3D), indicating a lack of RelA translocation and ablation of NF-κB signalling in a BLM dose-dependent manner. This resulted in reduced expression of multiple RelA target genes (such as IRF4, Bcl2, TNF, and CD40) in bone marrow and spleen cells of HKO and KO mice compared to WT (Fig. 2C, Figure. S3E). Additionally, the serum levels of NF-κB-regulated interleukins and chemokines (IL-2, CXCL2, CCL2, RANTES, IL-23, CXCL1) were decreased in HKO and KO mice compared to WT mice (Figure S3F).

To further elucidate the mechanism by which BLM regulates NF-κB signalling, we performed BLM ChIP-seq in FACS-sorted B220⁺ spleen B cells. Interestingly, in BLM ChIP-seq data, we found that BLM enriched on one of the critical regulators of NF-κB signalling, i.e., MALT1 (Fig. 2D). MALT1, an essential component of the CBM (CARMA1–BCL10–MALT1) complex [36]. ChIP-seq analysis revealed two BLM binding sites (site #1 and site #2) on the murine MALT1 promoter, indicating the regions of BLM occupancy (Fig. 2E, top panel). While BLM binding was detected at only site #2 in WT mice, its recruitment progressively diminished in HKO and KO mice (Fig. 2E). Consequently, we observed a progressive decline in *MALT1* transcript and protein levels in cells from HKO and KO mice (Figs. 2F, G), indicating that BLM directly regulates *MALT1* expression. This observation was also echoed in the analysis of scRNA-seq data from pro-B cells (Figure S4). The cleavage of multiple MALT1 substrates, including HOIL1 and BCL10, was also reduced in cells from BLM knockout (KO) mice (Fig. 2G). Together, the results indicate that the loss of NF-κB signalling in the absence of BLM leads to decreased proliferation and increased apoptosis, culminating in the inability of B cells to carry out normal development and differentiation.

### Re-expression of BLM, MALT1 and constitutively active IKKβ restores B-cell development in BLM KO mice

**Fig. 3:**
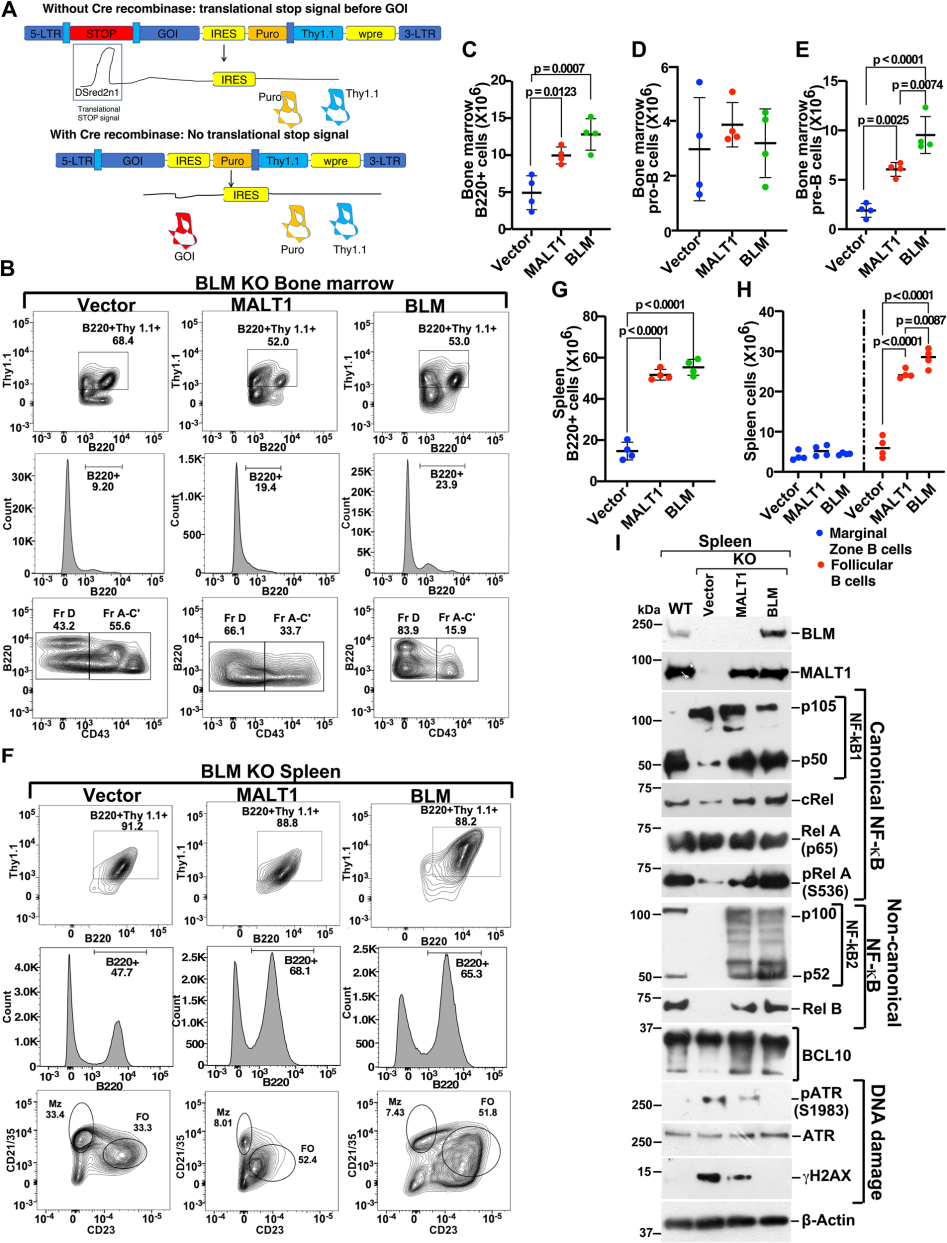
Rescue of B cell development in BLM-deficient mice by MALT1 and BLM. **A** *Schematic representation of the Cre-inducible retroviral vector system*. (Top) In the absence of Cre recombinase, a translational STOP cassette containing DSred2n1 prevents expression of the gene of interest (GOI). Only downstream elements, including puromycin resistance (Puro) and Thy1.1, are translated via the internal ribosome entry site (IRES). (Bottom) Upon Cre-mediated excision of the STOP cassette, the GOI is expressed along with the downstream Puro and Thy1.1 markers. The vector contains 5’ and 3’ long terminal repeats (LTRs), an IRES for bicistronic expression, and a woodchuck posttranscriptional regulatory element (wpre) for enhanced expression. **B** *Rescue of bone marrow B cell development in BLM-deficient mice by MALT1 and BLM*. Flow cytometric analysis of bone marrow cells from 12-week-old BLM KO mice transduced with retrovirus expressing either empty vector, MALT1, or BLM. Representative plots show marker expression, and the percentages of cells within the respective gates are indicated. Thy1.1 expression in bone marrow B cells is shown in the top row, total bone marrow cells and B220+ are shown in the middle row, and B220 + CD43+ (Fraction A-C’/ pro-B), B220 + CD43- (Fraction D/ pre-B) cells are shown in the bottom row. **C**–**E** *Both BLM and MALT1 rescue B-cell development*. Absolute numbers of bone marrow of (**C**) B220 + , (**D**) pro-B, (**E**) pre-B cells. Data is displayed as scatter plots, with each dot representing an individual mouse and horizontal bars denoting the mean for each group. **F** *Rescue of spleen-B cell development in BLM-deficient mice by MALT1 and BLM*. Same as (**B**) except spleen B-cell development was analyzed. Thy1.1 expression in bone marrow B cells is shown in the top row, total spleen cells and B220+ are shown in the middle row, and B220+CD23highCD21/35 + FO (Follicular B cells) and CD19+CD23lowCD21high MZ (Marginal zone B cells) are shown in the bottom row. **G**, **H** *Both BLM and MALT1 rescue follicular B cells in the spleen*. Absolute numbers of (**G**) spleen B220+ and (**H**) marginal zone and follicular B cells. Data is displayed as scatter plots, with each dot representing an individual mouse and horizontal bars denoting the mean for each group. **I** *NF-κB pathways were reactivated upon expressing MALT1 and BLM in BLM KO mice*. Western blot analysis was performed using whole-cell extracts from FACS-sorted spleen B220⁺ cells of WT, KO mice expressing either empty vector, MALT1 or BLM. Blots were probed with the indicated antibodies. Representative data for one biological replicate. Three biological replicates were done (1 mouse per genotype per biological replicate).

To assess whether expression of BLM or MALT1 can rescue B cell development in BLM- deficient mice, we transduced BLM knockout (KO) mice with a Cre-inducible retrovirus (Fig. 3A) encoding either an empty vector, MALT1 or BLM. Transgene expression was restricted to B cells via CD79a-Cre expression in the mice. Transduction efficiency was evaluated by measuring Thy1.1 expression in B cells. Transduction efficiency exceeded 50% in the B220+ cells of bone marrow (Fig. 3B, Top) and surpassed 80% in the B220+ cells of spleen (Fig. 3F, Top). MALT1 expression in BLM-deficient mice significantly increased total B cell numbers and improved the transition from pro-B to pre-B cells. However, this transition was 70%-75% in contrast to the rescue by BLM overexpression, thereby supporting BLM’s role in B cell development (Figs. 3B–E). Similarly, both BLM and MALT1 expression rescued splenic B cell development, restoring both total B cell and follicular B cell populations to comparable levels (Fig. 3F–H). Western blot analysis of splenic B cells of KO mice transduced with MALT1 or BLM showed restoration of both canonical and non-canonical NF-κB signalling pathways. BLM and MALT1-mediated rescue were evidenced by enhanced processing of NF-κB2 and NF-κB1, as well as increased expression of pRelA, c-Rel, and RelB. Notably, reintroduction of BLM also restored endogenous MALT1 levels (indicating that BLM is a direct upstream regulator of MALT1 expression) and diminished residual DNA damage in B cells by reducing the ATR-dependent DNA damage response to a basal level (Fig. 3I).

**Fig. 4:**
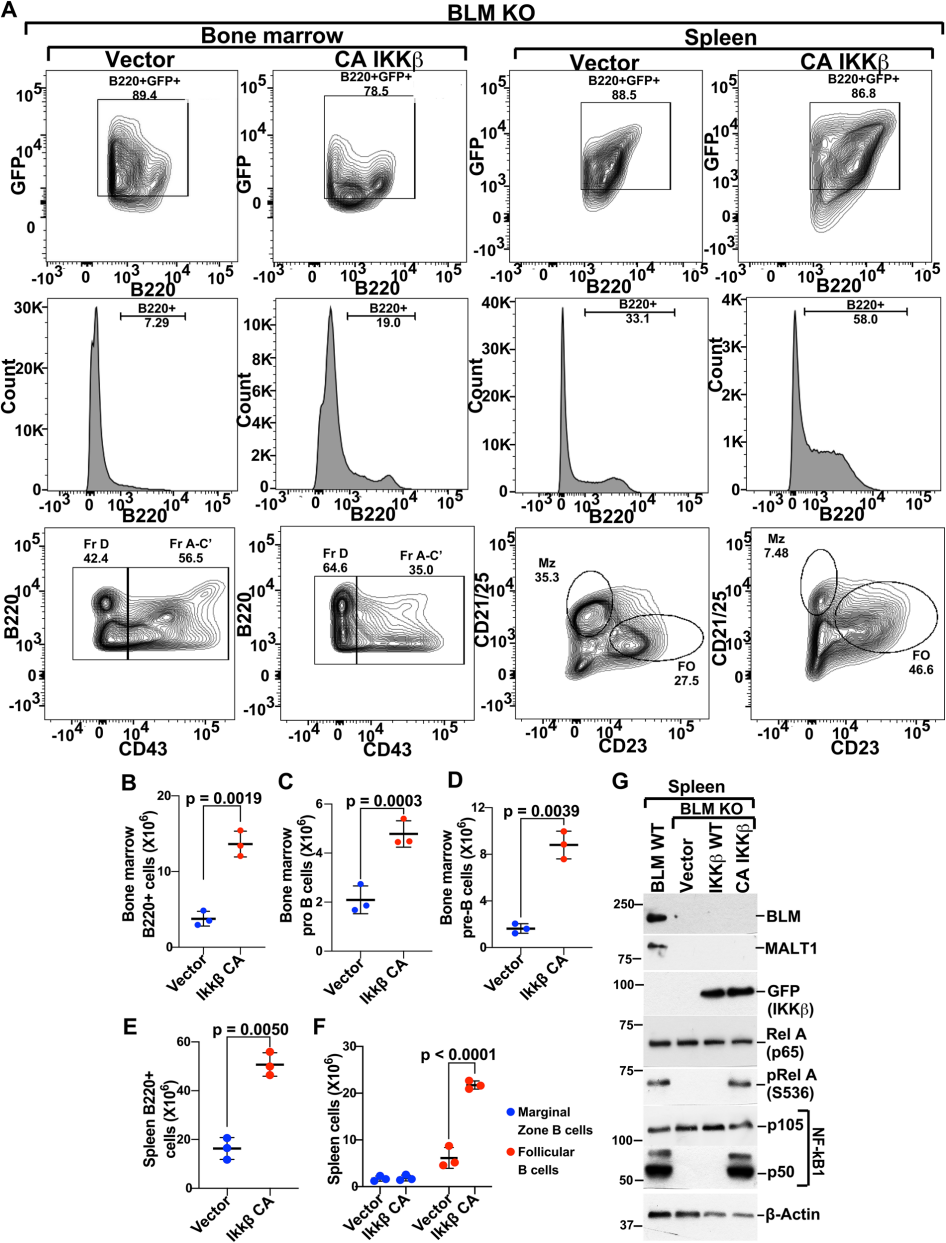
Rescue of B cell development in BLM-deficient mice by constitutively active (CA) IKKβ. **A** *Rescue of bone marrow and spleen B cell development in BLM-deficient mice by constitutively active IKKβ*. Flow cytometric analysis of bone marrow cells from 12-week-old BLM KO mice transduced with retrovirus expressing either empty vector, WT IKKβ, or CA IKKβ. Representative plots show marker expression, and the percentages of cells within the respective gates are indicated. (Left panel) GFP expression in bone marrow B cells is shown in the top row, total bone marrow cells and B220^+^ are shown in the middle row, and B220^+^CD43^+^ (Fraction A–C’/ pro-B), B220^+^CD43^-^ (Fraction D/ pre-B) cells are shown in the bottom row of panels. (Right panel) GFP expression in spleen B cells is shown in the top row, total spleen cells and B220^+^ are shown in the middle row, and B220^+^CD23^high^CD21/35^+^ FO (Follicular B cells) and CD19^+^CD23^low^CD21^high^ MZ (Marginal zone B cells) are shown in the bottom row. **B**–**D** *Constitutively active IKKβ rescues bone marrow B-cell development*. Absolute numbers of bone marrow of (**B**) B220^+^, (**C**) pro-B, (**D**) pre-B cells. Data is displayed as scatter plots, with each dot representing an individual mouse and horizontal bars denoting the mean for each group. **E**, **F** *Constitutively active IKKβ rescues follicular B cells in the spleen*. Absolute numbers of (**E**) spleen B220^+^ and (**F**) marginal zone and follicular B cells. Data is displayed as scatter plots, with each dot representing an individual mouse and horizontal bars denoting the mean for each group. **G** *NF-κB pathways were reactivated upon expressing constitutively active IKKβ in BLM KO mice*. Western blot analysis was performed using whole-cell extracts from FACS-sorted spleen B220⁺ cells of WT, KO mice expressing either empty vector, WT IKKβ, or CA IKKβ. Blots were probed with the indicated antibodies. Representative data for one biological replicate. Three biological replicates were done (1 mouse per genotype per biological replicate).

Since the CBM complex (CARMA1–BCL10–MALT1) activates the IKK complex, composed of IKKα (IKK1), IKKβ (IKK2), and NEMO (IKKγ) [37], activation of the IKK complex occurs through phosphorylation. For instance, IKKβ is activated by phosphorylation at S177 and S181 in mice, which subsequently phosphorylates IκBα, leading to its degradation and thereby promoting activation of canonical NF-κB signalling [38, 39]. To determine whether MALT1 downregulation is the primary cause of the NF-κB signalling defect in BLM-deficient cells, we performed a rescue experiment in BLM-KO cells using a constitutively active (CA) IKKβ mutant (IKKβ S177E, S181E). BLM-KO mice were transduced with a retrovirus encoding vector control, WT IKKβ, or CA IKKβ. Transduction efficiency was assessed by GFP expression, which ranged from 75–90% in B220⁺ cells from the bone marrow and spleen (Fig. 4A, top panel). Expression of CA IKKβ in BLM-deficient mice significantly increased total B cell numbers in the bone marrow and promoted the transition from pro-B to pre-B cells (Fig. 4A left; B–D). Similarly, CA IKKβ expression rescued splenic B cell development, restoring both total B cell and follicular B cell populations (Fig. 4A right; E, F). Furthermore, western blot analysis of splenic B cells from KO mice transduced with CA IKKβ showed restoration of canonical NF-κB signalling even in the absence of MALT1 (Fig. 4G), indicating that MALT1 downregulation is a primary contributor to the NF-κB signalling defect observed in BLM-deficient cells.

### BLM regulates MALT1 expression in human B cells

**Fig. 5:**
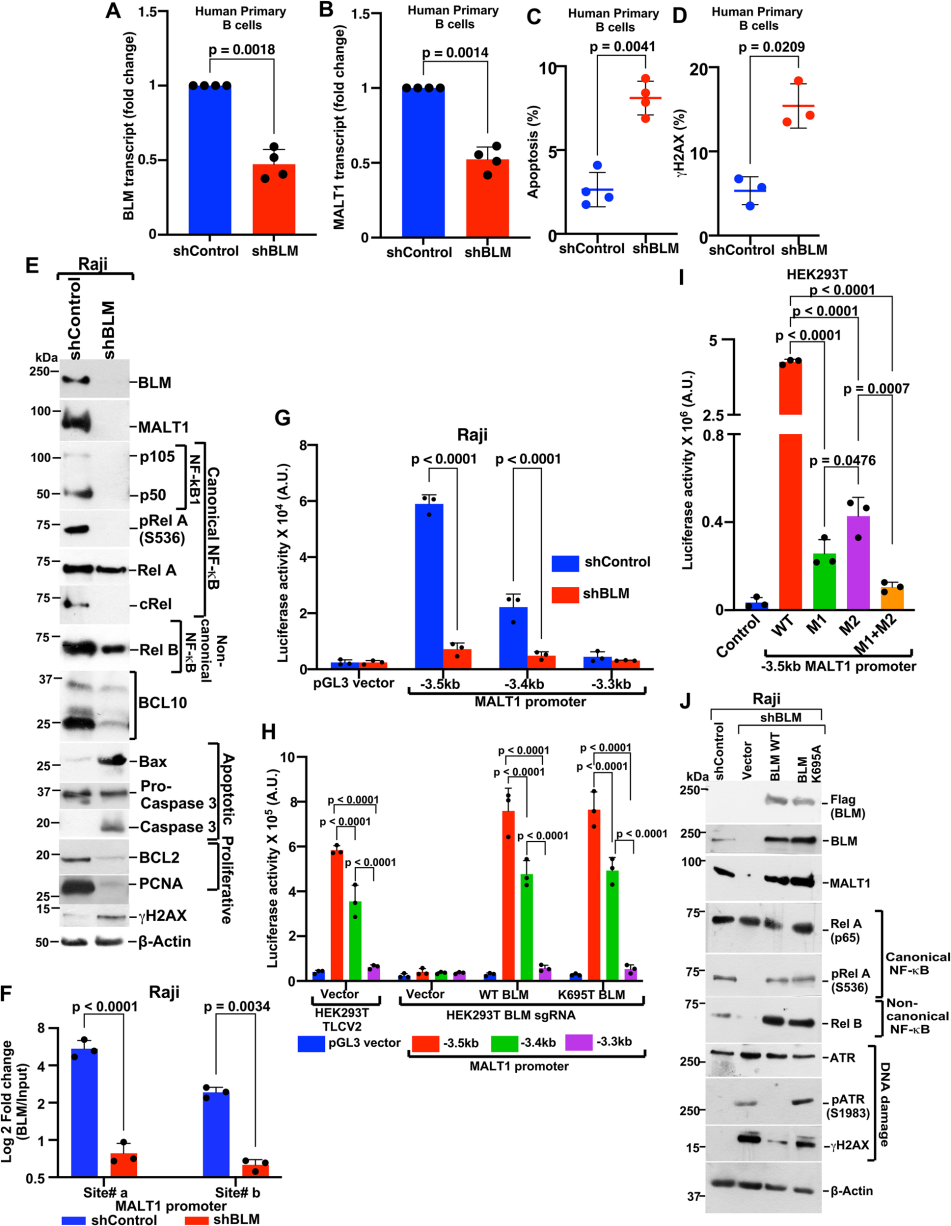
Effect of BLM ablation in human primary B cells, leukaemia and lymphoma cells. **A**, **B** *BLM depletion downregulates MALT1 levels in human primary B cells*. CD19^+^ cells (primary B cells) were isolated from human PBMCs. RNA was isolated from primary B cells transduced with either shControl or shBLM. RT-qPCR analysis of (**A**) BLM and (**B**) MALT1 was performed, and cortactin was used as an internal control. Data represent four biological replicates (1 human per biological replicate). Mean ± SD. **C**, **D** *BLM depletion enhances genome instability and apoptosis*. CD19^+^ cells (primary B cells) were isolated from human PBMCs transduced with either shControl or shBLM. **C** γH2AX staining was performed, and the percentage of γH2AX+ cells was plotted. Data represent three biological replicates (1 human per biological replicate). **D** Apoptosis assay was performed, and the percentage of AAD^+^ apoptotic cells was plotted. Data represent four biological replicates (1 human per biological replicate). Mean ± SD. **E** *BLM depletion impairs NF-κB signalling and promotes apoptosis in lymphoma cells*. Cell lysates were prepared from Raji cells stably expressing either shControl or shBLM. Western blot analysis was performed using the whole-cell extracts. Blots were probed with indicated antibodies to assess changes in NF-κB signalling and apoptotic markers. All experiments were independently repeated three times, and one representative result is shown. **F** *BLM recruits to the human MALT1 promoter*. Extent of recruitment of BLM to site #a (−3313 to −3437 w.r.t TSS), site #b (−3251 to −3376 w.r.t TSS) on the MALT1 promoter was determined by ChIP-qPCR using shControl and shBLM in Raji cells. Data represent three biological replicates. Mean ± SD. **G** *BLM regulates MALT1 promoter activity*. Basal MALT1 promoter activity was determined in Raji cells expressing shControl and shBLM by luciferase assays after co-transfecting the cells with either −3489 bp (having site #a, site #b), or −3346bp (having site #b), or −3280bp (having no sites) pGL3-MALT1 promoter constructs w.r.t TSS and CMV-β-galactosidase. A.U. is absolute units after normalization with β-galactosidase activity. Mean ± SD. The data is from three biological replicates. **H** *MALT1 promoter activity is independent of BLM helicase activity*. Basal MALT1 promoter activity was determined in HEK293T TLCV2 and HEK293T BLM sgRNA cells by luciferase assays after co-transfecting the cells with either −3489 bp (having site #a, #b), or −3346bp (having site #b), or −3280bp (having no sites) pGL3-MALT1 constructs, wild-type BLM, or helicase dead BLM (K695T) and CMV-β-galactosidase (acting as an internal control). A.U. is absolute units after normalization with β-galactosidase activity. Mean ± SD. The data is from three biological replicates. **I** *BLM binding sites on MALT1 promoter*. BLM binding sites on MALT1 promoters was determined in HEK293T cells by luciferase assays after co-transfecting the cells with either -wild-type 3489 bp (having WT site #a, site #b), or M1 (having mutated site #a, WT site #b), or M2 (having WT site, mutated site #a, mutated site #b) or M1 + M2 (having both site #a, site #b mutated) pGL3-MALT1 constructs and CMV-β-galactosidase. A.U. is absolute units after normalization with β-galactosidase activity. Mean ± SD. The data is from three biological replicates. **J** *BLM activates NF-κB signalling independent of its helicase activity*. Cell lysates were prepared from Raji cells stably expressing either shControl or shBLM or shBLM cells expressing either wild-type BLM or helicase-dead BLM (K695A). Western blot analysis was performed using the whole-cell extracts. Blots were probed with indicated antibodies to assess changes in NF-κB signalling and DNA damage response. All experiments were independently repeated three times, and one representative result is shown.

To investigate whether BLM regulates MALT1 expression in human cells, we performed shRNA-mediated knockdown of BLM in primary human B cells. BLM knockdown efficiency ranged from ~40–60% (Fig. 5A), which resulted in ~50% reduction in MALT1 levels (Fig. 5B). In addition, BLM depletion in primary B cells led to increased apoptosis (Fig. 5C and Figure. S5A) and elevated DNA damage, as indicated by increased γH2AX levels (Fig. 5D and Figure. S5B), suggesting enhanced genomic instability.

Next, we wanted to determine the effect of BLM knockdown in human lymphoma and leukaemia cells. Interestingly, BLM silencing in both Raji (Burkitt lymphoma) and RCH-ACV (ALL) cell lines led to reduced MALT1 expression and decreased levels of proteins involved in both canonical and non-cannonical NF-κB–dependent signalling pathways (Fig. 5E and Figure. S5C). Loss of BLM also decreased the expression of the anti-apoptotic protein BCL2 and the proliferative marker PCNA, while increasing pro-apoptotic BAX levels and inducing caspase-3 cleavage—collectively indicating enhanced apoptosis (Fig. 5E and Figure. S5C).

Further, to identify BLM-binding sites on the human MALT1 promoter, we aligned the human MALT1 promoter sequence with previously identified BLM-binding sites on the mouse MALT1 promoter. Both binding sites were found to be perfectly conserved in the human promoter (Figure S6A and Table S6). We performed BLM ChIP in shRNA-mediated BLM knockdown in Raji and RCH-ACV cell lines. In shControl Raji and RCH-ACV cells, BLM was recruited to both binding sites on the MALT1 promoter, likely due to their close proximity (Fig. 5F, Figure. S5D, S6B). Similarly, in other lymphoma cell lines, i.e. NAMLWA and Daudi, BLM was recruited to both sites (Figure S5E, S5F). However, stronger enrichment was observed at site #a than at site #b in all the cases (Fig. 5F and Figure. S5D–S5F). In addition, luciferase assays were carried out on the MALT promoter in Raji expressing cells shControl or shBLM and also in isogenic HEK293T cells transduced with either BLM sgRNA or vector (TLCV2). We observed maximal promoter activity in the presence of BLM but not in the absence of BLM with the –3.5 kb construct (containing both binding sites), reduced activity with the –3.4 kb construct (containing one site), and no activity with the –3.3 kb construct (lacking both sites) (Figs. 5G, H and Figure. S6B). Deletion of either of the individual BLM binding sites on the MALT1 promoter led to a reduction in MALT1 promoter activity, with the maximum decrease observed when both sites were deleted (Fig. 5I, Figure. S6B), suggesting that both the BLM binding sites on the MALT1 promoter are essential. Thus, BLM acts as a positive regulator of B-cell survival by positively regulating NF-κB signalling.

Recent evidence suggests that BLM helicase activity may or may not be required for its functions. The helicase activity is not essential for the effect of BLM on chromatin remodelling [14], the recruitment of a colon-specific transcription factor CDX2 during colon cancer development [40] or the unfolding of G-quadruplex [41]. However, it was essential for its role in the recruitment of HR and NHEJ factors onto the chromatin during different phases of the cell cycle [5]. Hence, we next asked whether MALT1 activation and NF-κB regulation require BLM helicase activity. In BLM CRISPR-knockout HEK293T cells (HEK293T BLM sgRNA), we observed exogenous expression of either wild-type BLM or helicase-dead BLM (K695T) in BLM knockout cells enhanced −3.5 kb and −3kb MALT1 promoter activity to near equivalent levels (Fig. 5H). Treatment of Raji shControl and shBLM cells with a BLM helicase activity inhibitor (ML216) revealed a similar extent of MALT1 expression and RelA activation in Raji shControl cells even after ML216 treatment (Figure S5G). Consequently, reconstitution of Raji cells lacking BLM with either wild-type or helicase-dead BLM restored NF-κB pathway activity and MALT1 activation (Fig. 5J). Notably, this rescue occurred despite persistent DNA damage in cells expressing the helicase-dead mutant, as indicated by elevated γH2AX and phospho ATR levels (Fig. 5J). These findings suggest that NF-κB and MALT1 regulation by BLM occurs independently of its helicase activity and is not a consequence of DNA damage accumulation.

### BLM inhibition enhances apoptosis and chemosensitivity in malignant B cells

**Fig. 6:**
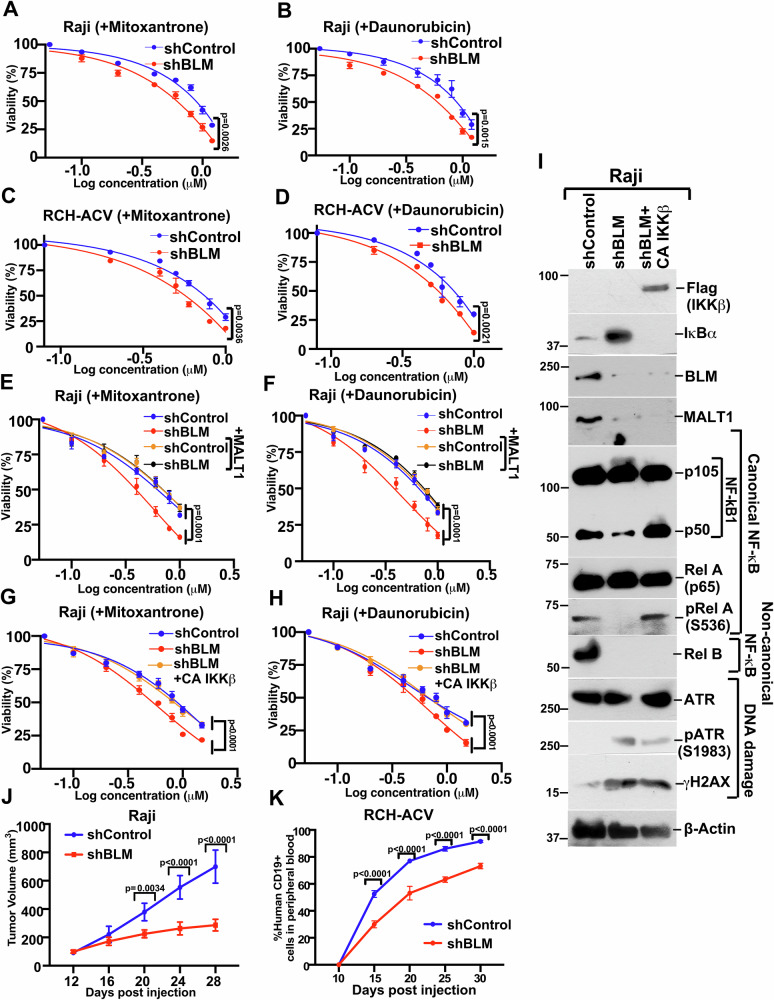
BLM-mediated MALT1 inhibition sensitizes human leukaemia and lymphoma cells. **A**–**D** *BLM depletion sensitizes lymphoma and leukaemia cells to chemotherapeutic agents*. **A**, **B** Raji or (**C**, **D**) RCH-ACV cells expressing either shControl or shBLM were treated with increasing concentrations of (**A**, **B**) Mitoxantrone and (**C**, **D**) Daunorubicin. After 36 h of drug exposure, cell viability was assessed using MTT assays. The data is from three biological replicates. Mean ± S.D. **E**, **F** *MALT1 inhibition sensitizes lymphoma and leukaemia cells to chemotherapeutic agents*. Raji cells expressing either shControl or shBLM or shControl expressing MALT1 or shBLM-expressing MALT1 were treated with increasing concentrations of (**E**) Mitoxantrone and (**F**) Daunorubicin. After 36 h of drug exposure, cell viability was assessed using MTT assays. The data is from three biological replicates. Mean ± S.D. *Constitutive active (CA) IKKβ in the absence of BLM desensitises lymphoma and leukaemia cells to chemotherapeutic agents*. Raji cells expressing either shControl or shBLM or shBLM-expressing CA IKKβ were treated with increasing concentrations of (**G**) Mitoxantrone and (**H**) Daunorubicin. After 36 h of drug exposure, cell viability was assessed using MTT assays. The data is from three biological replicates. Mean ± S.D. **I** *Constitutive active (CA) IKKβ in the absence of BLM activates NF-kB signalling*. Cell lysates were prepared from Raji cells stably expressing either shControl or shBLM or shBLM cells expressing CA IKKβ. Western blot analysis was performed using the whole-cell extracts. Blots were probed with indicated antibodies to assess changes in NF-κB signalling and DNA damage response. All experiments were independently repeated three times, and one representative result is shown. **J** *BLM inhibition decreases the rate of growth of xenografted Raji cells*. Tumors were generated by xenograft using shControl Raji and shBLM Raji cells, which were injected into NSG mice (*n* = 4). Tumor growth was measured for the indicated days. Mean ± SD. **K** Loss of BLM reduces the efficiency of RCH-ACV engraftment. NSG mice (*n* = 4) were injected with either shControl or shBLM RCH-ACV cells. The proportion of human CD19+ cells in peripheral blood was assessed by flow cytometry at the indicated time points. Data represent Mean ± SD.

NF-κB activation is a hallmark of several lymphoid and leukaemia malignancies [42, 43], and NF-κB inhibition is a major therapeutic approach in these malignancies [44, 45]. MALT1 inhibition has been shown to be effective in treating B cell malignancies by suppressing NF-κB signalling [46, 47]. Consistent with this, we also demonstrate that treatment with a MALT1 inhibitor (MI-2) led to NF-κB pathway inactivation and a corresponding decrease in cell viability (Figure S7A, S7B). Given that B-cell malignancies are driven by dysregulated B-cell proliferation, we next hypothesised that therapeutic inhibition of BLM could provide dual benefit by simultaneously suppressing MALT1-driven NF-κB signalling and impairing DNA damage repair, thereby improving treatment strategies for lymphoma and leukaemia. To test whether BLM silencing enhances sensitivity to standard chemotherapeutic agents used in these malignancies, we treated isogenic shControl and shBLM Raji and RCH-ACV cells with increasing concentrations of mitoxantrone and daunorubicin. In both cell lines, BLM knockdown markedly increased sensitivity compared to corresponding shControl cells (Fig. 6A–D and Table S7). To determine whether this chemosensitization results from BLM loss itself or from BLM-mediated repression of MALT1, we carried out lentiviral-mediated MALT1 expression in shControl and shBLM Raji cells and assessed their drug responses. Upon treatment, shBLM cells were chemosensitive, whereas shBLM cells expressing MALT1 became chemoresistant (Figs. 6E, F), thereby indicating that reduced MALT1 levels contribute significantly to the enhanced drug sensitivity.

To further determine whether BLM depletion causes MALT1 downregulation, which resulted in the impairment of the NF-κB proliferative pathway, rescue experiments were performed in Raji shBLM cells by constitutively expressing an active version of IKKβ (CA IKKβ). This resulted in the shBLM-expressing Raji cells showing restored cell viability and becoming chemoresistant (Fig. 6G, H). Additionally, CA IKKβ expression rescued the expression of key proteins in canonical NF-kB signalling pathways, even in the absence of MALT1 (Fig. 6I). Importantly, this rescue occurred despite persistent DNA damage, as γH2AX levels remained elevated in shBLM cells both with and without CA IKKβ expression (Fig. 6I). Based on the above cumulative results, it was hypothesized that shutdown or inhibition of BLM can be used as a therapeutic target for in vivo. Xenograft studies carried out using Raji cells in cells expressing or lacking BLM. Cells lacking BLM showed significantly reduced tumor growth compared to their BLM-expressing counterparts (Fig. 6J). Similarly, BLM depletion compromised the engraftment efficiency of RCH-ACV cells (Fig. 6K). Thus, the lack of BLM suppresses lymphoma and leukaemia by downregulating MALT1-dependent NF-κB signalling pathways.

## Discussion

BS patients are immunodeficient and exhibit a broad spectrum of phenotypes, highlighting the pleiotropic functions of BLM [48]. Notably, complete loss of BLM results in embryonic lethality in mice, emphasizing its essential role during development [49]. Although the importance of BLM in B cell lineage development has been previously recognized, [13], the regulatory mechanisms driving this phenotype remain unclear.

**Fig. 7:**
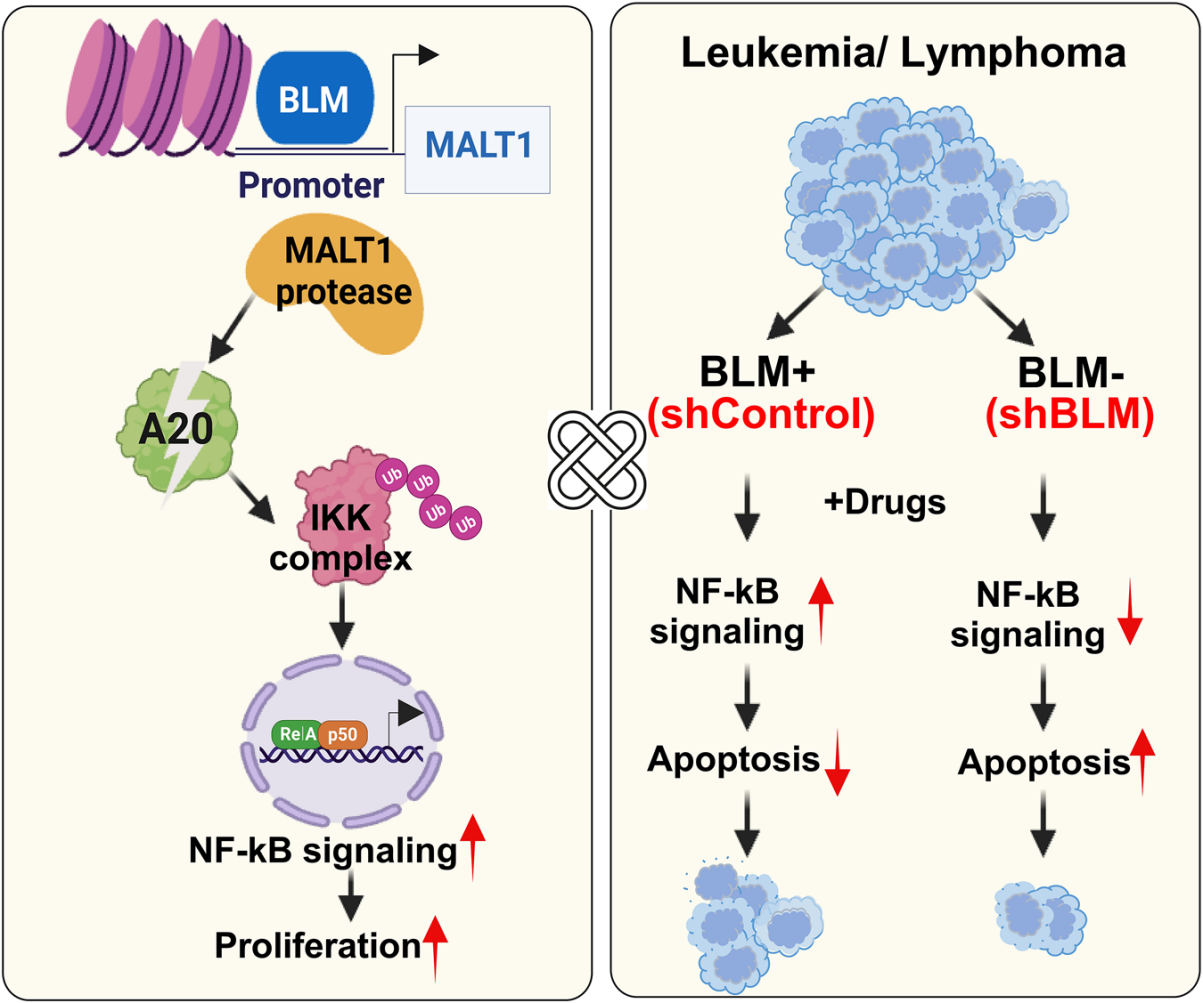
Schematic diagram illustrating the interconnective role of BLM in B cell development and B cell malignancies. Left panel: BLM binds to the procaspase MALT1 promoter and enhances its transcription, thereby enhancing its protease activity critical for cleaving and inactivating negative regulators of NF-κB signalling, such as A20, thereby activating the pathway. MALT1 contributes to the ubiquitination and subsequent degradation of IκBα, an inhibitor of NF-κB, thereby enhancing the translocation of the RelA/p50 NF-κB dimer into the nucleus and activating transcription of NF-κB target genes involved in cell proliferation and survival. BLM deficiency impairs this pathway, leading to reduced NF-κB signalling and proliferation. Right panel: BLM ablation chemosensitizes lymphoma and leukaemia cells to frontline chemotherapeutic drugs by downregulating NF-κB signalling. This reduction in NF-κB activity promotes apoptosis in response to chemotherapeutic drugs like Mitoxantrone and Daunorubicin.

Loss of BLM results in a marked reduction of the pre-B cell population, pointing to a disruption in the transition from pro-B to pre-B cells. This developmental block may be attributed to the accumulation of unrepaired DNA damage, leading to increased apoptosis and genomic instability. However, although precursor B cells proliferate normally, their subsequent maturation is impaired. This suggests that BLM deficiency disrupts the proliferative signals in later-stage B cells, potentially due to compromised NF-κB signalling. BLM influences NF-κB activity via positive transcriptional regulation of MALT1, which is not dependent on its helicase activity (Fig. 7, left). MALT1, in turn, orchestrates NF-κB signalling through both the canonical and non-canonical pathways [22, 50–52]. Hence, the lack or inhibition of BLM disrupts the progression from the pro-B to the pre-B stage via NF-κB-dependent process, ultimately impairing the maturation of functional B cells.

BLM is highly expressed in lymphoid organs such as the spleen and thymus [49]. Our data further reveal that BLM expression is elevated in cycling/proliferating B cells within the bone marrow and spleen. Importantly, loss of BLM results in a substantial reduction in these cycling B cell populations, thereby impairing clonal expansion. Thus, these findings emphasize the critical role of BLM in promoting B-cell proliferation and maintaining immune homeostasis. This is in line with clinical observations in BS patients, who exhibit markedly diminished levels of IgM, IgA and IgG [15–17]. Incidentally, 53BP1 is known to play a key role in NHEJ, CSR and V(D)J. Moreover, BLM is crucial for the recruitment of several NHEJ factors, including 53BP1 [24], XRCC4 [5], which is a key component of NHEJ, CSR, and V(D)J recombination [53, 54].

Paradoxically, BLM exhibits a dosage-sensitive duality in cancer cells, functioning as both a tumor suppressor and a potential oncogene in a context-dependent manner [4]. While BLM loss promotes genomic instability and tumorigenesis, excessive BLM expression can similarly drive malignant transformation [4]. Thus, alterations in BLM—whether reduced, elevated, or mutational—underscore its complex and context-dependent role during oncogenesis. Moreover, NF-κB activation is widespread in cancers, particularly during tumor initiation, metastasis, and the acquisition of therapeutic resistance [55]. Consequently, multiple therapies have been developed to target the NF-κB pathway, which remains a compelling therapeutic avenue [56]. Thus, inhibiting NF-κB signalling via BLM ablation in lymphoma and leukaemia may offer a precise and targeted therapeutic potential (Fig. 7, right). Importantly, the sensitization seen with BLM inhibition depends on MALT1, as restoring MALT1 or CA IKKβ expression in the absence of BLM does not confer chemosensitivity.

It will also be pertinent to mention that MALT1 deficiency phenocopies several aspects of BLM deficiency, including impaired B cell differentiation, agammaglobulinemia, and reduced peripheral B cell numbers [57], further supporting a functional link between the BLM–MALT1 axis in B cell biology and lymphomagenesis. Thus, inhibitors of BLM, which lead to its degradation (possibly through PROTAC-based design), should enhance the sensitivity of known chemotherapeutic drugs for lymphoma and leukaemia, and thereby act as a viable treatment option. Furthermore, suppression of MALT1, a critical mediator whose overexpression or hyperactivation is associated with specific B-cell lymphoma subtypes and immune dysfunction, is also a desirable option for treating both leukaemia and lymphoma [58, 59]. These findings collectively underscore the potential of targeting the BLM-MALT1 axis in the NF-κB pathway, which should help not only restore immune balance in B-cell malignancies but also provide therapeutic benefits to patients.

## Supplementary information

Supplimentary Information

CDDIS-25-7559

## Data Availability

The datasets produced in this study are available in the following database: Role of BLM helicase in B cells (scRNA seq): Gene Expression in Array Express (accession no. E-MTAB- 15173). https://www.ebi.ac.uk/biostudies/ArrayExpress/studies/E-MTAB-15173?key=bde8806e- 1917-469b-aeb9-b980b9cd4e44. The datasets produced in this study are available in the following database: Role of BLM helicase in B cells (ChIP seq): Gene Expression in Array Express (accession no. E-MTAB- 15120). https://www.ebi.ac.uk/biostudies/ArrayExpress/studies/E-MTAB-15120?key=d6b6570f-1f26- 4d04-b611-a7ac1946e0a2.
