# Supplementary material for: BLM regulates MALT1-driven NF-κB signalling and is targetable in B-cell malignancies
Source: Cell Death Dis. 2026 May 20;17(1):636. doi: 10.1038/s41419-026-08846-3 (PMC13358038; doi:10.1038/s41419-026-08846-3)
Supplement: Supplementary file 1 — Supplimentary Information [file 41419_2026_8846_MOESM1_ESM.docx]

SUPPLEMENTARY INFORMATION

**BLM regulates MALT1-driven NF-κB signalling and is targetable in B-cell malignancies**

Ritu Agrawal^1,2, *^, Supratim Ghosh^2^ Nitin Kumar^1^, Chetana Mukherjee^1^, Vandana Sharma^1^, Rimpy Arun^1^, Riya Deb^2^, Sumanta Sarkar^2^, Savita^1^, Dilip Kumar^2^, Satyajit Rath^3^, Arindam Maitra^2, *^, Sagar Sengupta^1,2, *^

**Affiliations:**

1 Biotechnology Research and Innovation Council—National Institute of Immunology (BRIC- NII), Aruna Asaf Ali Marg, New Delhi, India.

2Biotechnology Research and Innovation Council—National Institute of Biomedical Genomics (BRIC-NIBMG), Kalyani, India.

3Indian Institute of Science Education and Research, Dr Homi Bhabha Road, Pashan, Pune, Maharashtra, India.

***Corresponding authors:**

Sagar Sengupta : [ssg2@nibmg.ac.in](mailto:ssg2@nibmg.ac.in)

Arindam Maitra : [am1@nibmg.ac.in](mailto:am1@nibmg.ac.in)

Ritu Agrawal : [rituagrawal@nii.ac.in](mailto:rituagrawal@nii.ac.in)

**Keywords:** BLM, B cell development, NF-κB pathway, MALT1, Lymphoma, Leukaemia

**This file includes:**

Supplementary Material & Method

Supplementary Figure S1 to S7

Supplementary Tables S1 to S6

#
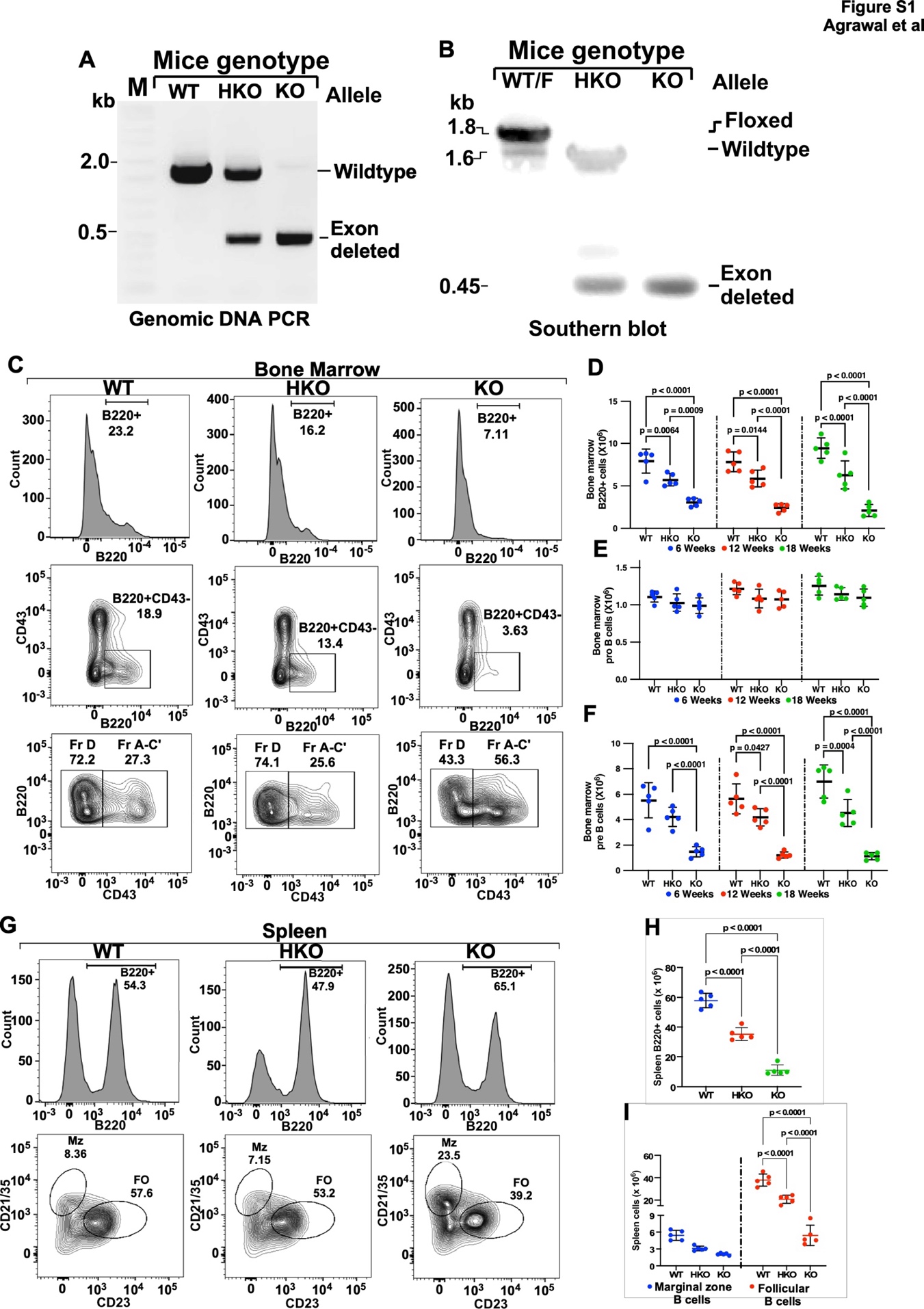


**Figure S1 legend:**

**A, B. Screening of BLM genotype. DNA was extracted from FACS/MACS-sorted B220+ B cells of the spleen.** BLM genotype of the mice - wildtype (WT), hetero knockout (HKO) and knockout (KO) mice were determined by (A) Genomic DNA PCR and (B) Southern blot analysis. Representative results are shown. (A) was done for every mouse used in the study, (B) n=4 mice.

**C-F. Defective bone marrow B cell development in BLM-deficient mice.** (C) Flow cytometric analysis of bone marrow cells from 6-, 12-, and 18-week-old BLM WT, HKO, and KO mice was performed for expression of the indicated markers, and percentages of cells within the respective gates were given. FACS data represented is from 12-week-old mice. Total bone marrow cells and B220+ are shown in the top row, and B220^+^CD43^+^ (Fraction A-C’/ pro-B) cells are shown in the middle row, B220^+^CD43^-^ (Fraction D/ pre-B) cells are in the bottom row of row. (D-F) Absolute numbers of bone marrow (D) B220^+^, (E) pro-B and (F) pre-B cells are displayed as scatter plots, with each dot representing an individual mouse and horizontal bars denoting the mean for each group. Quantified data is for 6-, 12- and 18-week-old mice.

**G-I. Defective spleen-B cell development in BLM-deficient mice.** (G) Flow cytometric analysis of spleen cells from 12-week-old mice of BLM WT, HKO and KO mice was analysed for expression of the indicated markers, and percentages of cells within the respective gates were given. Total spleen cells and B220+ are shown in the top row, and B220^+^CD23^high^CD21/35^+^ FO (Follicular B cells) and CD19^+^CD23^low^CD21^high^ MZ (Marginal zone B cells) in the bottom row. (H, I) Absolute numbers of (H) spleen B220^+^ and (I) marginal zone and follicular B cells are displayed as scatter plots, with each dot representing an individual mouse and horizontal bars denoting the mean for each group.

**
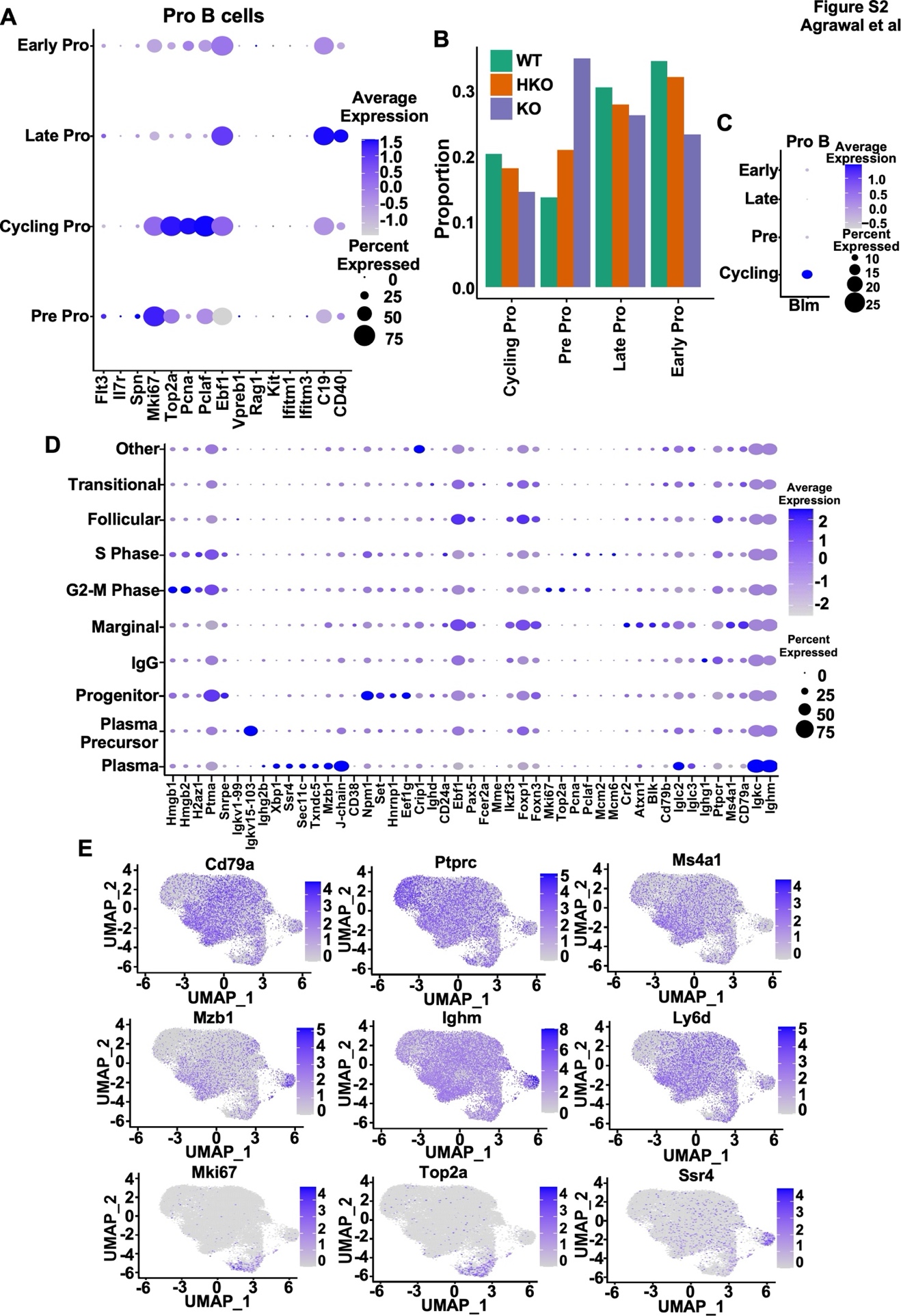
**

**Figure S2 legend:**

**A, D. Expression of lineage and subset-specific genes across pro-B cell and spleen-B cell subpopulations.** Dot plot depicting expression of (A) pro-B cell (D) spleen-B cell subtype-specific marker genes used for cell state annotation. The dot size indicates the percentage of cells expressing each gene, while the intensity of the dot colour represents the average expression level.

**B. Proportional distribution of pro-B cell subpopulations across genotypes.** Bar plots showing the relative abundance of pro-B cell subpopulations across all three genotypes.

**C. BLM expression enriched in cycling pro-B cell subsets.** Same as (A) except the dot plot illustrating the expression of BLM in different pro-B subpopulations of WT cells.

**E. Expression levels of key markers in spleen B cells.** Feature plot showing expression of pan B cell and B cell state-specific marker genes in the spleen B cell UMAP space. Colour intensity represents the average expression level.

**
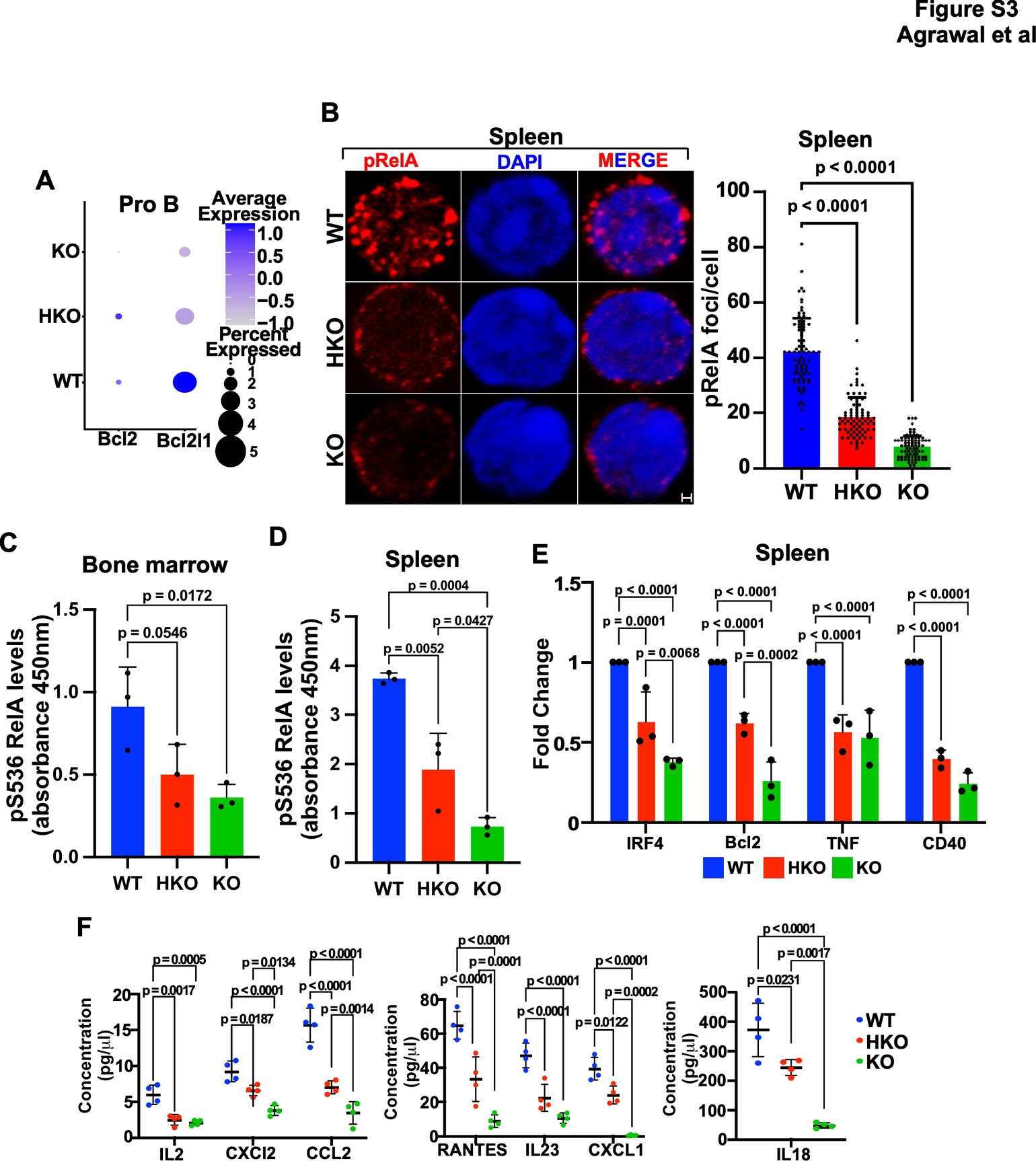
**

**Figure S3 legend:**

**A. Anti-apoptotic NF-κB targets were reduced in BLM KO mic**e. Dot plot illustrating the expression of Bcl2 and Bcl2l1 in pro-B cells across all three genotypes. The dot size indicates the percentage of cells expressing each gene, while the intensity of the dot colour represents the average expression level.

**B. Phospho-RelA levels were reduced in the absence of BLM in the spleen B-cells.** (Left) Immunofluorescence was carried out on FACS-sorted B220⁺ cells from spleen using antibodies against phospho RelA (S536). DNA was counterstained with DAPI. Bar: 5μm. (Right) Quantitation of immunofluorescence carried out in spleen cells. Number of nuclei analysed in each case (n ≥80) from three biological replicates, n = 1 mouse per genotype per biological replicate. Mean ± SD.

**C, D. Phospho RelA levels are enhanced in the presence of BLM as determined by ELISA.** ELISA-based quantification of pRelA (S536) levels was carried out using whole cell lysates of FACS-sorted B220⁺ cells of (C) bone marrow and (D) spleen, using an NF-κB p65 (pS536) ELISA assay. Absorbance at 450 nm was measured. Data represent three biological replicates. n=1 mouse per genotype per biological replicate. Mean ± SD.

**E. RelA targets are transcriptionally downregulated in BLM HKO and KO mice.** RNA was isolated from FACS-sorted B220+ B cells from the spleen. RT-qPCR analysis of the indicated RelA targets was performed across all three genotypes. Data represent three biological replicates (1 mouse per genotype per biological replicate). Mean ± SD.

**F. Reduced NF-κB-regulated interleukins and chemokines in BLM-deficient mice.** Indicated interleukins and chemokines were quantified using ProcartaPlex™ multiplex immunoassay in serum samples from BLM WT, HKO and KO mice. n=4 mice. Mean ± SD.

**
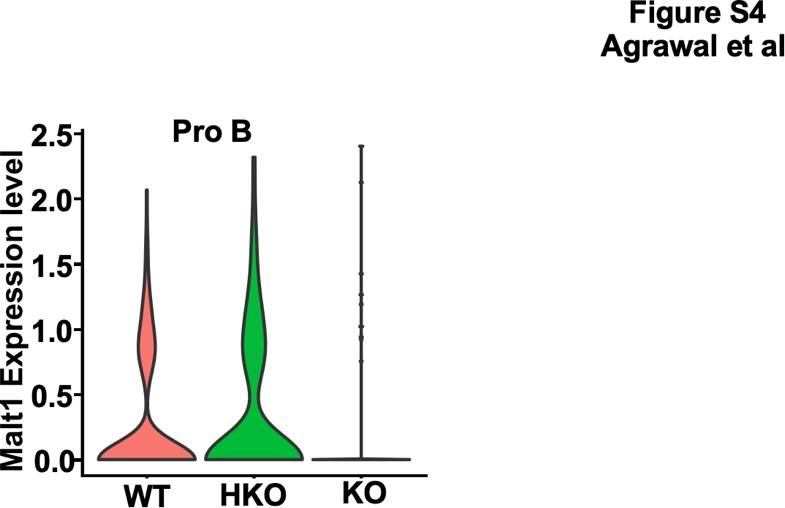
**

**Figure S4 legend:**

**Malt1 transcript reduced in BLM KO mice.** Violin plot illustrating the expression of *Malt1* in pro-B cells across all three genotypes. Each violin represents the distribution of single-cell expression values.

**
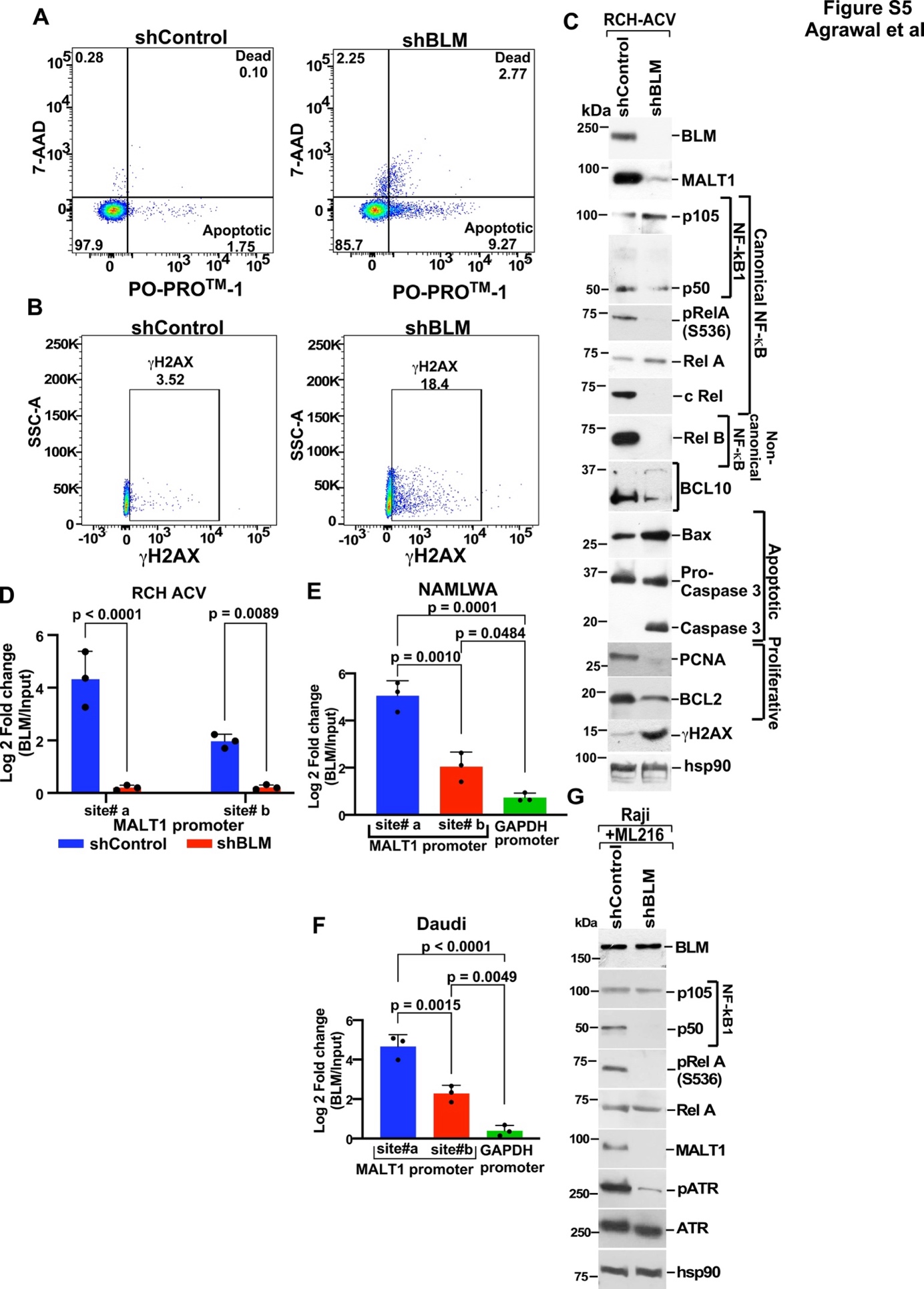
**

**Figure S5 legends:**

**A, B. BLM depletion enhances apoptosis and genome instability.** CD19^+^ cells (primary B cells) were isolated from human PBMCs transduced with either shControl or shBLM. (A) Apoptosis was assessed by FACS analysis. The experiment included four biological replicates (one human donor per biological replicate), and one representative result is shown. (B) γH2AX staining was performed to assess genome instability, and cells were analyzed by FACS. The experiment included three biological replicates (one human donor per biological replicate), and one representative result is shown.

**C. BLM depletion impairs NF-κB signaling and promotes apoptosis in leukaemia cells.** Cell lysates were prepared from RCH-ACV cells stably expressing either shControl or shBLM. Western blot analysis was performed using the whole-cell extracts. Blots were probed with indicated antibodies to assess changes in NF-κB signalling and apoptotic markers. All experiments were independently repeated three times, and one representative result is shown.

**D-F. BLM recruits to the human MALT1 promoter.** Extent of recruitment of BLM to site #a (-3313 to -3437 w.r.t TSS), site #b (-3251 to -3376 w.r.t TSS) on the MALT1 promoter was determined by ChIP-qPCR using (D) shControl and shBLM in RCH-ACV (E) NAMLWA (F) Daudi cells. Data represent three biological replicates. Mean ± SD.

**G. MALT1 activation does not depend on BLM helicase activity.** Cell lysates were prepared from Raji cells stably expressing either shControl or shBLM and treated with 12.5μM ML216 for 24 h. Western blot analysis was performed using the whole-cell extracts. Blots were probed with the indicated antibodies. All experiments were independently repeated three times, and one representative result is shown.

#
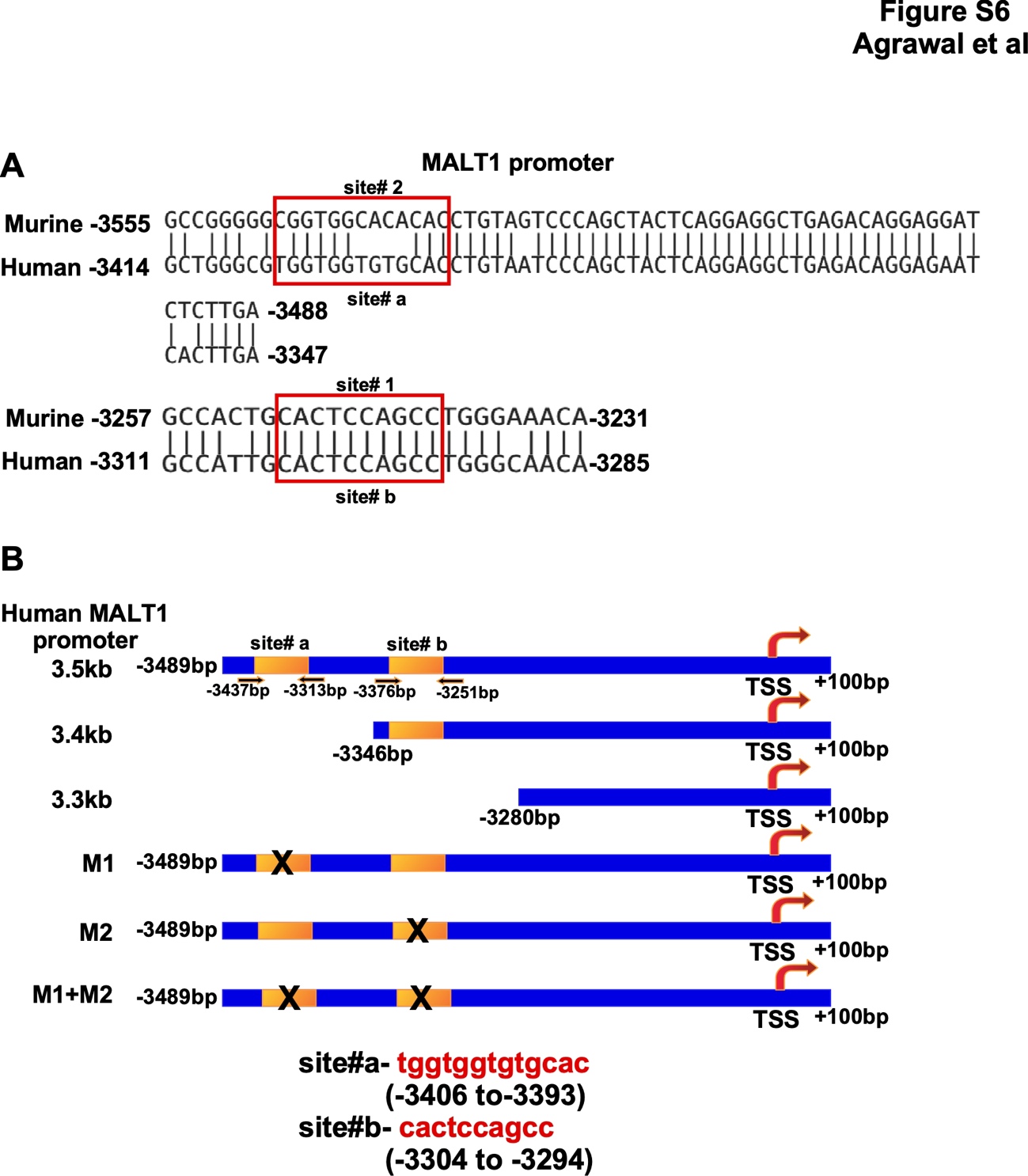


# Figure S6 legend:

# A. Conservation of BLM binding sites between the mouse and human MALT1 promoters. The sequences in the murine MALT1 promoter were aligned with the human MALT1 promoter. The corresponding conserved binding sites for BLM are highlighted in a red box. BLM binding sites in the murine promoter are shown by site #1 and site #2, while their counterparts in the murine promoter are denoted as site #1 and site #b.

**B.** **Schematic representation of the human MALT1 promoter showing the positions of the BLM binding sites.** The promoter length relative to the TSS is indicated on the left. The 3.5 kb MALT1 promoter in human contains both binding sites (#a and #b). The large arrow indicates the TSS, and small arrows indicate the positions of the ChIP–qPCR primer binding sites. The 3.4 kb promoter contains only site #b, while the 3.3 kb promoter lacks both sites. M1 represents the mutation of site #a with site #b remaining wild type, M2 represents the mutation of site #b with site #a remaining wild type, and M1+M2 indicates that both sites are mutated.

**
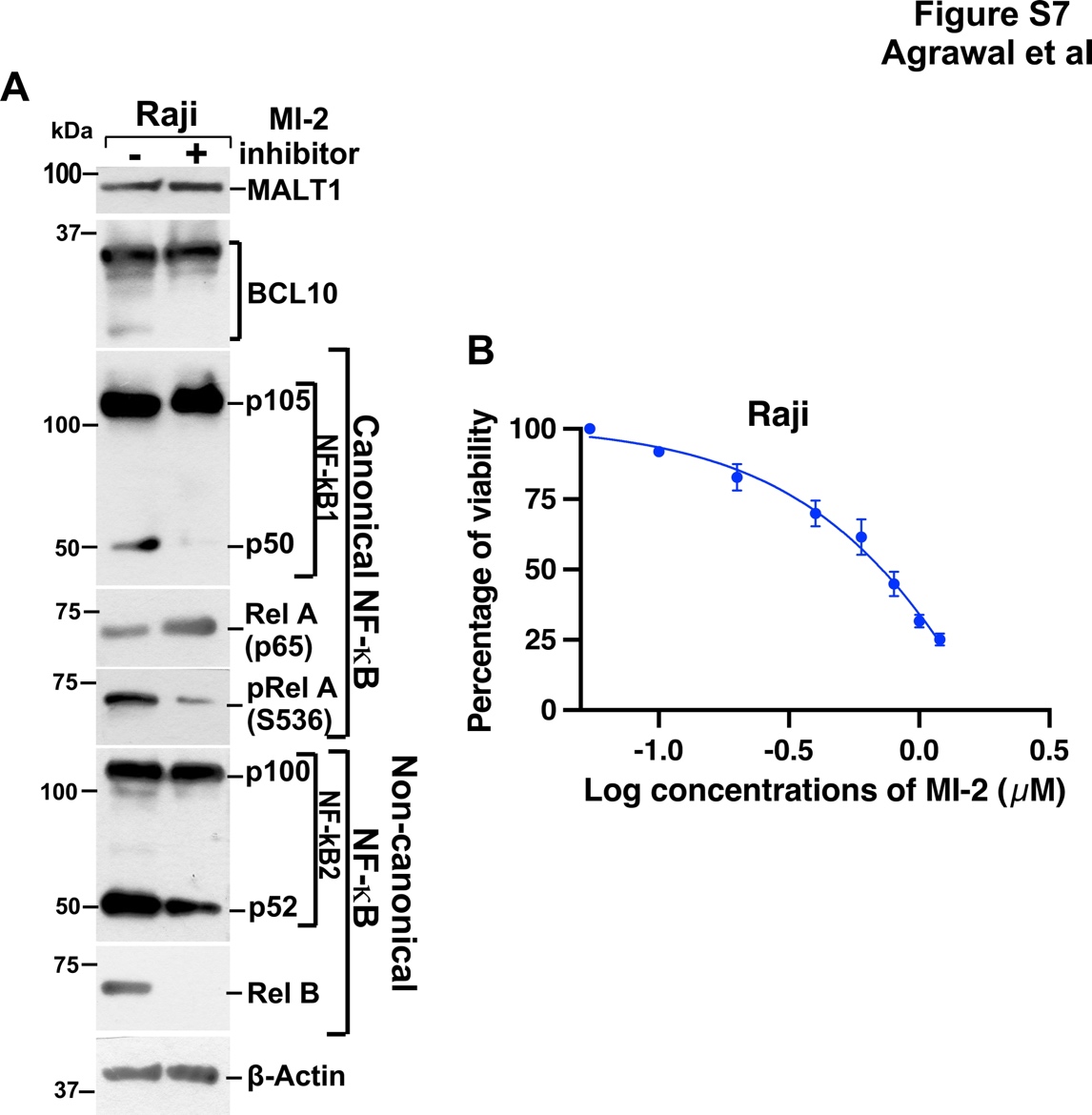
**

**Figure S7 legend:**

**A. MALT1 inhibition inhibits NF-kB signalling in lymphoma cells.** Cell lysates were prepared from Raji cells treated with 0.1μM MI-2 (MALT1 inhibitor for its protease activity) for 24 hours. Western blot analysis was performed using the whole-cell extracts. Blots were probed with the indicated antibodies. All experiments were independently repeated three times, and one representative result is shown.

**B.** **MALT1 inhibition reduces cell viability of lymphoma cells. Raji cells** were treated with increasing concentrations of MI-2 inhibitor. After 24 hours of drug exposure, cell viability was assessed using MTT assays. The data is from three biological replicates. Mean ± S.D.

**Table S1: List of antibodies used in the study**

| **Name of antibody** | **Source** | **Identifier (Dilution used)** |
| --- | --- | --- |
| Anti-BLM  (Used for WB, ChIP seq, ChIP) | Bethyl Laboratories | Cat#A300572A; RRID:  AB_669671  (WB: 1:1000, ChIP and ChIP seq:  3μg/reaction) |
| Anti-NFκB1 (p105/p50) (Used for WB) | Cell Signalling | Cat#13586; RRID: AB_2665516 (WB: 1:1000) |
| Anti-RelA (p65)  (Used for WB) | Bethyl Laboratories | Cat# 3033; RRID: AB_1264341 (WB: 1:1000) |
| Anti-p-NFκB p65 (S536) or Anti-pRelA (Used for IF) | Cell Signalling | Cat#3033; RRID: AB_331284 (IF: 1:200) |
| Anti-RelB (Used for WB) | Cell Signalling | Cat#4922; RRID: AB_2179173 (WB: 1:1000) |
| Anti-cRel (Used for WB) | Santa Cruz Biotechnology | Cat#sc-71; RRID: AB_2253705 (WB: 1:1000) |
| Anti-NFκB2 (Used for WB) | Cell Signalling | Cat#37359; RRID: AB_2799114 (WB: 1:1500) |
| Anti-IkBα (Used for WB) | Cell Signalling | Cat#4812; RRID: AB_10694416 (WB: 1:1500) |
| Anti-p53 (Used for WB) | Oncogene | Cat#OP03-100UG; RRID: AB_10690076 (WB: 1:1000) |
| Anti-ATR (Used for WB) | Santa Cruz Biotechnology | Cat#; sc515173; RRID:  AB_289329 (WB: 1:1000) |
| Anti-pATR (T1983)  (Used for WB) | GeneTex | Cat#GTX128145; RRID: AB_2687562  (WB: 1:2000) |
| Anti- β Actin (Used for WB) | Santa Cruz Biotechnology | Cat# sc47778; RRID: AB_626632 (WB: 1:2000) |
| Anti-hsp90 (Used for WB) | Santa Cruz Biotechnology | Cat#sc69703; RRID:  AB_2121191(WB: 1:2000) |
| Anti-Histone H3 (Used for WB) | Abcam | Cat# ab1791, RRID: AB_302613 (WB: 1:1000) |
| Anti-MALT1 (Used for WB) | Cell Signaling Technology | Cat# 2494, RRID: AB_2139139 (WB: 1:1000) |
| Anti-BCL10 (Used for WB) | Santa Cruz Biotechnology | Cat# sc-5273, RRID: AB_626730 (WB: 1:2500) |
| Anti-RBCK1 (HOIL1)  (Used for WB) | Santa Cruz Biotechnology | Cat# sc-365523, RRID: AB_10841591 (WB: 1:1000) |
| Anti-Bcl2 (Used for WB) | Santa Cruz Biotechnology | Cat# sc-492, RRID: AB_465151 (WB 1:3000) |
| Anti-Bax (Used for WB) | Santa Cruz Biotechnology | Cat# sc-493, RRID: AB_2227995 (WB 1:3000) |
| Anti-Caspase 3 (Used for WB) | Santa Cruz Biotechnology | Cat# sc-7148, RRID: AB_637828 (WB: 1:3000) |
| Anti-Flag  (Used for WB) | Merck | Cat# F3165, RRID: AB_259529  (WB: 1:2000) |
| Anti-GFP  (Used for WB) | Santa Cruz Biotechnology | Cat# sc-8334, RRID: AB_641123  (WB: 1:2000) |
| Anti-yH2AX  (Used for WB) | Abcam | Cat# ab26350, RRID: AB_470861  (WB: 1:3000) |
| Anti-PCNA  (Used for WB) | Santa Cruz Biotechnology | Cat# sc-56, RRID: AB_628110  (WB: 1:3000) |
| PE mouse Anti-Human γH2AX  (Used for FC) | Thermo Fisher Scientific | Cat#12-9865-42, RRID:AB_2572753  (FC: 1:200) |
| APC Rat Anti-Mouse IgM (Used for FC) | BD Biosciences | Cat#550676, RRID: AB_398464 (FC: 1:1000) |
| V450 Rat anti-Mouse CD45R (Used for FC) | BD Biosciences | Cat#560472, RRID: AB_1645276 (FC: 1:1000) |
| PE Rat Anti-Mouse CD43 (Used for FC) | BD Biosciences | Cat#553271, RRID: AB_394748 (FC: 1:1000) |
| PE-Cy™7 Rat Anti-Mouse CD45R/B220  (Used for FC) | BD Biosciences | Cat#552772, RRID: AB_394458 (FC: 1:1500) |
| PE Rat Anti-Mouse CD23 (Used for FC) | BD Biosciences | Cat# 553139, RRID: AB_394654 (FC: 1:1000) |
| FITC Rat Anti-Mouse CD21/CD35  (Used for FC) | BD Biosciences | Cat# 561769, RRID:  AB_10924591 (FC: 1:1000) |
| FITC Rat Anti-Mouse CD90.1 (Thy1.1)  (Used for FC) | Thermo Fisher Scientific | Cat#11-0900-81, RRID: AB_465151 (FC: 1:2000) |
| TruStain FcX™ PLUS (anti- mouse CD16/32) Antibody | BioLegend | Cat# 156603, RRID: AB_2783137 |
| **Hashtags** | | |
| TotalSeq™-C0301 anti-mouse Hashtag 1 Antibody (ACCCACCAGTAAGAC) | BioLegend | Cat# 155861, RRID: AB_2800693 |
| TotalSeq™-C0302 anti-mouse Hashtag 2 Antibody (GGTCGAGAGCATTCA) | BioLegend | Cat# 155863, RRID: AB_2800694 |
| TotalSeq™-C0303 anti-mouse Hashtag 3 Antibody (CTTGCCGCATGTCAT) | BioLegend | Cat# 155865, RRID: AB_2800695 |

WB: Western blotting

ChIP seq: Chromatin Immunoprecipitation Sequencing IF: Immunofluorescence

FC: Flow Cytometry

**Table S2: List of recombinant DNAs used in the study.**

| **Name of the recombinant DNA** | **Source** | **Identifier** |
| --- | --- | --- |
| pMSCV-loxP-dsRed-loxP- eGFP-Puro-WPRE | Hans Clevers (University Medical Centre, Utrecht, Netherlands) | Addgene Cat#32702 |
| plenti-CAG-gate-FLAG-IRES- GFP plasmid | William Kaelin (Department of Medical Oncology, Dana- Farber Cancer Institute, Boston, MA, USA) | Addgene Cat#107398 |
| pCL-Eco | Inder Verma (Laboratory of Genetics, The Salk Institute, San Diego, California, USA) | Addgene Cat#12371 |
| MSCV-LoxP-DSRed2N1-LoxP- DEST-IRES-Puro-T2A-Thy1.1 | This study | N/A |
| MSCV-TraSTOP-GFP-IRES-  Puro-T2A-Thy1.1 | This study | N/A |
| MSCV-TraSTOP-murine-BLM- IRES-Puro-T2A-Thy1.1 | This study | N/A |
| MSCV-TraSTOP-murine MALT1-IRES-Puro-T2A-Thy1.1 | This study | N/A |
| pLVX-IRES-Hygro-Flag MALT1 | This study | N/A |
| pLVX-IRES-Hygro-Flag BLM | This study | N/A |
| pLVX-IRES-Hygro-Flag BLM (K695A) | This study | N/A |
| pGL3 MALT1 promoter (-3489bp to +100bp w.r.t TSS) | This study | N/A |
| pGL3 MALT1 promoter (-3346bp to +100bp w.r.t TSS) | This study | N/A |
| pGL3 MALT1 promoter (-3280bp to +100bp w.r.t TSS) | This study | N/A |
| pGL3 MALT1 promoter (-3489bp to +100bp w.r.t TSS) with site #a mutated indicated as M1 | This study | N/A |
| pGL3 MALT1 promoter (-3489bp to +100bp w.r.t TSS) with site #b mutated indicated as M2 | This study | N/A |
| pGL3 MALT1 promoter (-3489bp to +100bp w.r.t TSS) with site #a and site #b mutated indicated as M1+M2 | This study | N/A |
| pBABE FLAG-IKK2-EE puro (human CA IKKβ) | Kevin Janes (University of Virginia, Charlottesville, USA) | Addgene Cat# 216790  (1) |
| retro-gfpIkkb-puro vector  (mice WT IKKβ) | Georgios T. Stathopoulos  (Faculty of Medicine, University of Patras, Rio, Greece) | Addgene Cat# 58251  **(2)** |
| retro-gfpIkkb-puro vector  (S177E, S181E)  (mice CA IKKb) | This study | N/A |
| pENTR^TM^1A | Invitrogen | Cat# A10462 |
| pLKO.1 puro shControl | Present in the lab | N/A |
| pLKO.1 puro shBLM | Present in the lab | N/A |
| pMD2.G/V-SVG | Didier Trono (School of Life Sciences, Ecole Polytechnique Fédérale de Lausanne, Switzerland) | Addgene Cat#12259 |
| pxPAX2 | Didier Trono (School of Life Sciences, Ecole Polytechnique Fédérale de Lausanne, Switzerland) | Addgene Cat#12260 |

**Table S3: List of reagents used in the study**

| **Names** | **Source** | **Identifier** |
| --- | --- | --- |
| **Chemicals** | | |
| Tween 20 | Merck | Cat# P1379; CAS Number: 9005-64-5 |
| NaOH | Merck | Cat#106462; CAS Number: 1310-73-2 |
| NaCl | Merck | Cat#106404; CAS Number 7647-14-5 |
| Tris | Merck | Cat# 102408; CAS Number 77-86-1 |
| Triton X-100 | Merck | Cat# 108624; CAS Number 9036-19-5 |
| DMSO (100%) | Thermo Fisher Scientific | Cat#F515 |
| TRIzol Reagent | Thermo Fisher Scientific | Cat# 15596026 |
| Trypan Blue Solution | Merck | Cat# T8154 |
| Glycerol (99.5%) | Merck | Cat# G9012 |
| Ambion Nuclease Free Water | Invitrogen | Cat#AM9937 |
| SPRIselect | Beckman Coulter | Cat#B23318 |
| EB buffer | Qiagen | Cat#19086 |
| Absolute Ethanol | Merck | Cat#1.00983.0511 |
| Thiazolyl Blue Tetrazolium Bromide (MTT) | Merck | Cat# M2128; CAS Number 298-93-1 |
| Mitoxantrone dihydrochloride | Merck | Cat# M6545; CAS Number 70476-82-3 |
| Daunorubicin hydrochloride | Merck | Cat# 30450; CAS Number 23541-50-6 |
| Puromycin dihydrochloride | Merck | Cat# P9620; CAS Number 58-58-2 |
| Hygromycin B | Merck | Cat# 10843555001; CAS Number 31282-04-9 |
| ML216 | Merck | Cat#SML0661 |
| Formaldehyde solution | Merck | Cat# F8775; CAS Number 50-00-0 |
| Dynabeads™ CD19 Pan B | Thermo Fisher Scientific | Cat#11143D |
| DETACHaBEAD™ CD19 Kit | Thermo Fisher Scientific | Cat#12506D |
| MALT1 inhibitor MI-2 | MedChemExpress | Cat# HY-12276; CAS Number 1047953-91-2 |
| HiSep™ LSM 1077 (HIMEDIA: SKU: LS001) | HiMedia | Cat# LS001 |
| X-tremeGENE™ HP DNA | Roche | Cat# 06366236001 |
| Lipofectamine 3000 | Thermo Fisher Scientific | Cat# L3000015 |
| Lipofectamine 2000 | Thermo Fisher Scientific | Cat# 11668019 |
| **Kit** | | |
| Chromium Next GEM Chip K Single Cell Kit, 48rxns | 10X Genomics | Cat#1000286 |
| Chromium Next GEM Single Cell 5' Kit v2, 16 rxns | 10X Genomics | Cat#1000263 |
| Library Construction Kit, 16 rxns | 10X Genomics | Cat#1000190 |
| Dual Index Kit TT Set A, 96 rxns | 10X Genomics | Cat#1000215 |
| Dual Index Kit TN Set A, 96 rxns | 10X Genomics | Cat#1000250 |
| 5' Feature Barcode Kit | 10X Genomics | Cat#1000541 |
| Qubit dsDNA HS Assay Kit, 500 assays | Invitrogen | Cat# Q32854 |
| KAPA Library Quant Kit (Illumina) | Roche | Cat#7960204001 |
| NovaSeq 6000 S2 Reagent Kit v1.5 (100 cycles) | Illumina | Cat#20028316 |
| Reverse transcriptase core kit | Eurogentec | Cat# RT-RTCK-03 |
| DyNamo color flash SYBR green qPCR kit | Thermo Fisher Scientific | Cat# F-416L |
| True MicroChIP-seq Kit | Diagenode | Cat# C01010132 |
| NFkB p65 (pS536 + Total) Simple step ELISA Kit | Abcam | Cat#ab176663 |
| NE-PER™ Nuclear and Cytoplasmic Extraction kit | Thermo Fisher Scientific | Cat#78833 |
| PicoPure™ DNA Extraction Kit | Thermo Fisher Scientific | Cat#KIT0103 |
| Gateway™ LR Clonase™ II Enzyme mix | Thermo Fisher Scientific | Cat#11791020 |
| ProcartaPlex™ Mouse Immune Response Panel, 64plex | Thermo Fisher Scientific | Cat#EPX640-20064-901 |
| Membrane Permeability Dead Cell Apoptosis Kit | Thermo Fisher Scientific | Cat#V35123 |
| **Animals** | | |
| Blmtm4Ches/J (Referred as BLM fl/fl) | The Jackson Laboratory | Stock# 008670 |
| B6.C(Cg)-  Cd79atm1(cre)Reth/EhobJ (Referred as Cd79a Cre) | The Jackson Laboratory | Stock# 020505 |
| BLM wild-type mice referred as WT | This study | N/A |
| BLM hetroknockout mice referred as HKO | This study | N/A |
| BLM knockout mice referred as KO | This study | N/A |
| NSG mice | The Jackson Laboratory | Stock# 005557 |
| **Cell lines** | | |
| HEK293T | Present in the lab | ATCC Cat# CRL-3216 |
| Raji | Present in the lab | ATCC Cat# CCL-86 |
| RCH-ACV | Vaskar Saha (Tata Translational Cancer Research Centre, India) | Cellosaurus Cat#CVCL-1851 |
| Raji shControl | This study | N/A |
| Raji shBLM | This study | N/A |
| Raji shControl expressing MALT1 | This study | N/A |
| Raji shBLM expressing MALT1 | This study | N/A |
| RCH-ACV shControl | This study | N/A |
| RCH-ACV shBLM | This study | N/A |
| HEK293T | Present in the lab | (3) |
| HEK293T TLCV2 | Present in the lab | (3) |
| HEK293T BLM sgRNA | Present in the lab | (3) |
| NAMLWA | Present in the lab | N/A |
| Daudi | Present in the lab | N/A |
| **Others** | | |
| DPBS (1X) | Gibco | Cat#14190-144 |
| Fetal bovine Serum | Thermo Fisher Scientific | Cat# 10082147 |
| RPMI media | Thermo Fisher Scientific | Cat#21870076 |
| Cryostor CS10 Freezing Media | BioLife Solutions | Cat#210102 |
| 10XPBS | Merck | Cat# 79383 |
| Complete Protease Cocktail inhibitor | Roche | Cat# 11697498001 |
| Taq DNA Polymerase (3U/μl) | Geneilab | Cat#0601600051730 |
| Deoxynucleotide (dNTP) Solution Mix | New England Biolabs | Cat#N0447L |
| Taq Buffer A (Tris with 15 mM MgCl2) | Geneilab | Cat#0653100011730 |
| LongAmp® Taq DNA Polymerase | New England Biolabs | Cat#M0323L |
| CD45R (B220) MicroBeads, mouse | Milteny Biotec | Cat#130-049-501 |
| LS columns | Milteny Biotec | Cat#130-042-401 |
| **Software** | | |
| GraphPad Prism 9 | GraphPad | https://[www.graphpad.com/sc](http://www.graphpad.com/sc) ientific-software/prism/ |
| IgV tool | IgV | https://igv.org/app/ |
| Image J | Image J | <https://imagej.net/Fiji> |
| FlowJo | FlowJo | [https://www.flowjo.com/solut](https://www.flowjo.com/solutions/flowj/) [ions/flowj/](https://www.flowjo.com/solutions/flowj/) |
| RStudio | RStudio Team | <https://rstudio.com/> |
| **Deposited Data** |  |  |
| Raw sequencing reads for BLM ChIP-seq | Array Express | Accession number: E-MTAB-15120 |
| BLM ChIP-seq data analyzed (chromosomal location and TSS) |  |  |
| Raw sequencing reads and processed Seurat objects for scRNA seq | Array Express | Accession number: E-MTAB-15173 |

**Table S4: Primers used in the present study**

| **Genes** | **Forward Primer Sequence (5’-3’)** | **Reverse Primer Sequence (3’- 5’)** |
| --- | --- | --- |
| **RT-qPCR primers** | | |
| CD40 (murine) | ACCAGCAAGGATTGCGA GGCAT | GGATGACAGACGGTATCA GTGG |
| Bcl2 (murine) | TGAGTACCTGAACCGGC ATCT | GCATCCCAGCCTCCGTTAT |
| IRF4 (murine) | GCCCAACAAGCTAGAAA G | TCTCTGAGGGTCTGGAAA CT |
| TNF (murine) | TCTCTGAGGGTCTGGAAA CT | TCTCTGAGGGTCTGGAAA CT |
| MALT1 (murine) | GAACTGAGCGACTTCCTA CAGG | AACTGTCCAGCCAACACT GCCT |
| BLM (human) | AGACAGGATTCTCTGCCACCAGGA | TGGTGTTTCAGCCCAGTTGCT |
| MALT1 (human) | TGGAAGCCCTATTCCTCA CTACC | CATGACACCAGTAGGTTC CTTGG |
| Cort-actin (human) | GCCGACCGAGTAGACAAG | GTATTTGCCGCCGAAACC |
| β-actin (murine) | CAGCCTTCCTTCTTGGGT ATG | GGCATAGAGGTCTTTACG GATG |
| **ChIP qPCR primers** | | |
| MALT1 promoter site #1 (mice) | GGATCTCGCCTGTGAATAGTAG | CATCTCTCCAGCCCTTTCT TT |
| MALT1 promoter site #2  (mice) | TTAAGAAAAGCTAGGGT CGCC | GGACACCTGATCAGCATA GC |
| MALT1 promoter site #a (human) | TCTACTAAAAATACAAAATAT | GATCTCGGCTCACGGCAACCT |
| MALT1 promoter site #b  (human) | CAGGAGGCTGAGACAGGAGAA | TTAATTTATTTATTTTGAGAC |
| GAPDH promoter | GCAGCCCCTTCATACCCTCACGT | GAGCCACACCATCCTAGTTGC |
| **Genotyping PCR** | | |
| BLM (Jackson Primer) | AACCTGCTTCAGCTAGG AGCTTCC | TGGGACCGAATTGCTTCAA CAACG |
| CD79a Cre | CTCTTTACCTTCCAAFCA CTGA CATTTTCGAGGGAGCTTC A | ACTGAGGCAGGAGGATTG G |
| **Long Amp PCR** | | |
| BLM | ATTGCAGTTCCACTACTC CAAGT | ATTTAGGCTTCATTCTGAG G |
| **Cloning Primers** | | |
| pENTR^TM^1A BLM | CGGGGTACCCCGGCCAC CATGAGGATCATGGCTG CTGTT | CCGCTCGAGCGGTTAGGA GAAGGCATATGAAGG |
| pENTR^TM^1A MALT1 | CGGGGTACCCCGGCCAC CATGTCGCTGTGGGGGC AGCCG | CCGCTCGAGCGGTCAGTT TTCAGAAATCATAAG |
| pLVX-IRES-Hygro-Flag MALT1 | ATAAGAATGCGGCCGCTA AACTATGCCACCATGGACTACAAAGACCATGACGGTGATTATAAAGATCATGACATCGATTACAAGGATGACGAT GACAAGGAGGAGCCG CAGTCAGATCCT | CGCGGATCCGCGTCATTTTTCAGAAATTCTGAG |
| pGL3 MALT1 promoter (-3489bp to +100bp w.r.t TSS) | CGGGGTACCCCGCACCTGAGGTCAGGAGTTCAAGAGCAGCCTG | CTAGCTAGCTAGCGCCTCCGAGCCGCGGAGGCAGGGGCGGAAG |
| pGL3 MALT1 promoter (-3346bp to +100bp w.r.t TSS) | CGGGGTACCCCGCCTGGGAGGTGGAGGTTGCCGTGAGCCG | CTAGCTAGCTAGCGCCTCCGAGCCGCGGAGGCAGGGGCGGAAG |
| pGL3 MALT1 promoter (-3280bp to +100bp w.r.t TSS) | GGGGTACCCCATAACTCCGTCTCAAAATAAATAAATTAA T | CTAGCTAGCTAGCGCCTCCGAGCCGCGGAGGCAGGGGCGGAAG |
| **Oligos** | | |
| pLKO.1 puro shControl | Merck | Cat#SHC007 |
| pLKO.1 puro shBLM | CCGGGCCTTTATTCAATA CCCATTTCTCGAGAAAG GGTATTGAATAAAGGCT TTTTG | AATTCAAAAAGCCTTTAT TCAATACCCATTTCTCGA GAAATGGGTATTGAATAA AGGC |

**Table S5: Statistical analysis performed in this study**

| **Figure number** | **Statistical Analysis performed** | **Type of test** | **Software used** |
| --- | --- | --- | --- |
| Figure 2B  (Right) | Ordinary one-way ANOVA | Dunnett's multiple comparisons test | GraphPad Prism |
| Figure 2C | Two-way ANOVA | Holm-Šídák's multiple comparisons test | GraphPad Prism |
| Figure 2E | Two-way ANOVA | Šídák's multiple comparisons test | GraphPad Prism |
| Figure 2F | Ordinary one-way ANOVA | Tukey's multiple comparisons test | GraphPad Prism |
| Figure 3C | Ordinary one-way ANOVA | Tukey's multiple comparisons test | GraphPad Prism |
| Figure 3D | Ordinary one-way ANOVA | Tukey's multiple comparisons test | GraphPad Prism |
| Figure 3E | Ordinary one-way ANOVA | Tukey's multiple comparisons test | GraphPad Prism |
| Figure 3G | Ordinary one-way ANOVA | Tukey's multiple comparisons test | GraphPad Prism |
| Figure 3H | Two-way ANOVA | Tukey's multiple comparisons test | GraphPad Prism |
| Figure 4B | Paired T test | Two-tailed | GraphPad Prism |
| Figure 4C | Paired T test | Two-tailed | GraphPad Prism |
| Figure 4D | Paired T test | Two-tailed | GraphPad Prism |
| Figure 4E | Paired T test | Two-tailed | GraphPad Prism |
| Figure 4F | Two-way ANOVA | Šídák's multiple comparisons test | GraphPad Prism |
| Figure 5A | Paired T test | Two-tailed | GraphPad Prism |
| Figure 5B | Paired T test | Two-tailed | GraphPad Prism |
| Figure 5C | Paired T test | Two-tailed | GraphPad Prism |
| Figure 5D | Paired T test | Two-tailed | GraphPad Prism |
| Figure 5F | Two-way ANOVA | Šídák's multiple comparisons test | GraphPad Prism |
| Figure 5G | Two-way ANOVA | Šídák's multiple comparisons test | GraphPad Prism |
| Figure 5H | Two-way ANOVA | Tukey's multiple comparisons test | GraphPad Prism |
| Figure 5I | Ordinary one-way ANOVA | Tukey's multiple comparisons test | GraphPad Prism |
| Figure 6A-6H | Two-way ANOVA | Šídák's multiple comparisons test | GraphPad Prism |
| Figure 6B | Two-way ANOVA | Šídák's multiple comparisons test | GraphPad Prism |
| Figure 6J | Two-way ANOVA | Šídák's multiple comparisons test | GraphPad Prism |
| Figure 6K | Two-way ANOVA | Šídák's multiple comparisons test | GraphPad Prism |
| Figure S3B(Right) | Ordinary one-way ANOVA | Dunnett's multiple comparisons test | GraphPad Prism |
| Figure S3C | Ordinary one-way ANOVA | Dunnett's multiple comparisons test | GraphPad Prism |
| Figure S3D | Ordinary one-way ANOVA | Dunnett's multiple comparisons test | GraphPad Prism |
| Figure S3E | Two-way ANOVA | Tukey's multiple comparisons test | GraphPad Prism |
| Figure S3F(Left) | Two-way ANOVA | Bonferroni's multiple comparisons test | GraphPad Prism |
| Figure S3F (Middle) | Two-way ANOVA | Tukey's multiple comparisons test | GraphPad Prism |
| Figure S3F (Right) | Ordinary one-way ANOVA | Tukey's multiple comparisons test | GraphPad Prism |
| Figure S5D | Two-way ANOVA | Šídák's multiple comparisons test | GraphPad Prism |
| Figure S5E | Ordinary one-way ANOVA | Tukey's multiple comparisons test | GraphPad Prism |
| Figure S5F | Ordinary one-way ANOVA | Tukey's multiple comparisons test | GraphPad Prism |

**Table S6: BLM binding site on MALT1 promoter**

MALT1 mice promoter

**>-3600bp to -3180bp w.r.t TSS**

GTTTTTTTATTTGGTTATTTCGTCAGCTTAAGAAAAGCTAGGGTCGCCGGGGGCGGTGGC
ACACACCTGTAGTCCCAGCTACTCAGGAGGCTGAGACAGGAGGATCTCTTGAGTCCAGTT
CTGGTCTGTAGTGCGCTATGCTGATCAGGTGTCCGCACTAAGTTTGGCATTAATATGGTG
ATCTCTCTGGAGCGGGGGGGGGGGGTGGGGGGTGGGGGGGGGAGAACCACCAGGTTGTCT
AAGGAGGGGTGAACCGGCCCAGGTAGGAAACGGAGCAGGTCAAAACTCCCGTGTTGAACA
GTAGTGGGATCTCGCCTGTGAATAGTAGTAACAGTAGTGAATAGCCACTGCACTCCAGCC
TGGGAAACATAGTGAGACACCATCTCTTTTAAGAAAAAAAGAAAGGGCTGGAGAGATGAT

site #1: highlighted in green. site #2 : highlighted in pink

Human mice promoter

**> -4000bp to -3220bp w.r.t TSS**AGACATCGGGTTTGGTCAACATTGTATTCTGCAAACTCTAAGGGAAAACTGGTGAAGACC
GAACTGTGGTGTGGGAATCCCGCCTAGCTCATCTGCCTGGGTTGTCTTTATCTTTGTCTC
ATGACTAGGCTACTTTACAACTCTGAGATCGCAGTGAACTTACAGAAAACTGATAGTGAT
GCAAGTGAATCTTGTTTATAATTAAATAAATTGTCAGGTGGAGGTTCAATTGCATTCCCT
CCTCCACTGTGGAAGAAGCTAGTTTTGCATCTAGGAAGCAACTTCATTTCAGTGGGCTGA
CATATTTATCTGGTCTTTGGGTTCTCCTCTGAAGTAATTGTAACACCTTTCTGATTCCCT
TGTTAGTTAATGAGTTAAAATCTCTGATATGTGTTCATTGCATATTTGTTGCATCATGAT
TTAAACTTTCTTGTAGAAACTATTCTTAAGACCGGACGCGGTGGCTCATGCCTGTAATCC
CAGCACTTTGGGAGGGCCGACATGGGTGAATCACCTGAGGTCAGGAGTTCAAGAGCAGCC
TGGGCAACATGGTGAAACCCGTCTCTACTAAAAATACAAAATATTAGCTGGGCGTGGTGG
TGTGCACCTGTAATCCCAGCTACTCAGGAGGCTGAGACAGGAGAATCACTTGAACCTGGG
AGGTGGAGGTTGCCGTGAGCCGAGATCGTGCCATTGCACTCCAGCCTGGGCAACAAGAGC
ATAACTCCGTCTCAAAATAAATAAATTAATCAAATAAAAACTATTCTTTAACAGTATGTA

site #a: highlighted in pink. site #b: highlighted in green.

Highlighted in yellow + pink + green represent aligned sequence with Mice promoter

**Table S7: EC50 values of stably expressing shControl and shBLM -Raji and RCH-ACV upon treatment with either Mitoxantrone or Daunorubicin**

1. **Effect of Mitoxantrone on Raji**

|  | **Raji shControl** | **Raji shBLM** |
| --- | --- | --- |
| **EC50 (µM)** | 1.371 | 0.774 |
| **p value (t-test)** | 0.0026 | |

1. **Effect of Daunorubicin on Raji**

|  | **Raji shControl** | **Raji shBLM** |
| --- | --- | --- |
| **EC50 (µM)** | 1.565 | 0.7005 |
| **p value (t-test)** | 0.0015 | |

1. **Effect of Mitoxantrone on RCH-ACV**

|  | **RCH-ACV shControl** | **RCh-ACV shBLM** |
| --- | --- | --- |
| **EC50 (µM)** | 1.882 | 0.9348 |
| **p value (t-test)** | 0.0036 | |

1. **Effect of Daunorubicin on RCH-ACV**

|  | **RCH-ACV shControl** | **RCh-ACV shBLM** |
| --- | --- | --- |
| **EC50 (µM)** | 0.6676 | 0.4452 |
| **p value (t-test)** | 0.0021 | |

**References**

1. Griffiths CD, Shah M, Shao W, Borgman CA, Janes KA. Three modes of viral adaption by the heart. Sci Adv. 2024;10(46):eadp6303.

2. Giannou AD, Marazioti A, Spella M, Kanellakis NI, Apostolopoulou H, Psallidas I, et al. Mast cells mediate malignant pleural effusion formation. J Clin Invest. 2015;125(6):2317-34.

3. Agrawal R, Agarwal H, Mukherjee C, Chakraborty B, Sharma V, Tripathi V, et al. Phosphorylated BLM peptide acts as an agonist for DNA damage response. Nucleic Acids Res. 2025;53(4).
