# Supplementary material for: BLM regulates MALT1-driven NF-κB signalling and is targetable in B-cell malignancies
Source: Cell Death Dis. 2026 May 20;17(1):636. doi: 10.1038/s41419-026-08846-3 (PMC13358038; doi:10.1038/s41419-026-08846-3)
Supplement: Supplementary file 2 — CDDIS-25-7559 [file 41419_2026_8846_MOESM2_ESM.pdf]

Agrawal et al
Raw Data

Figure 2A

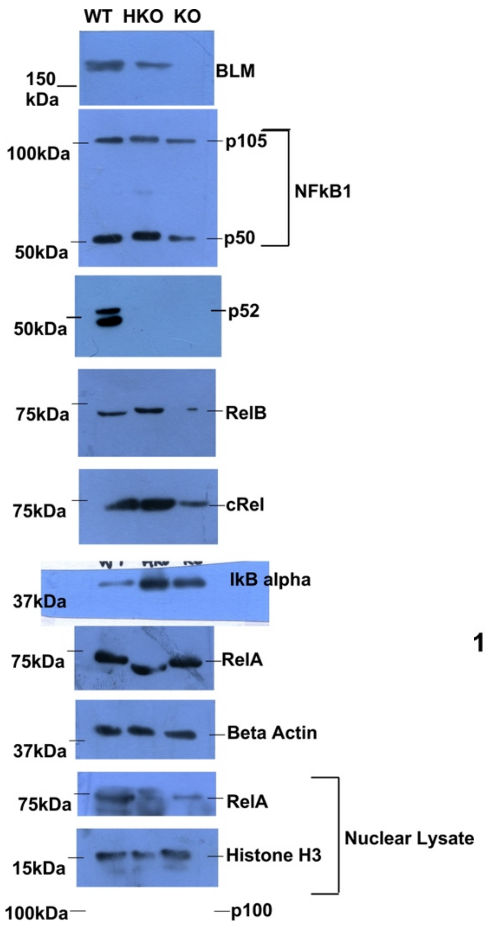

Figure2G

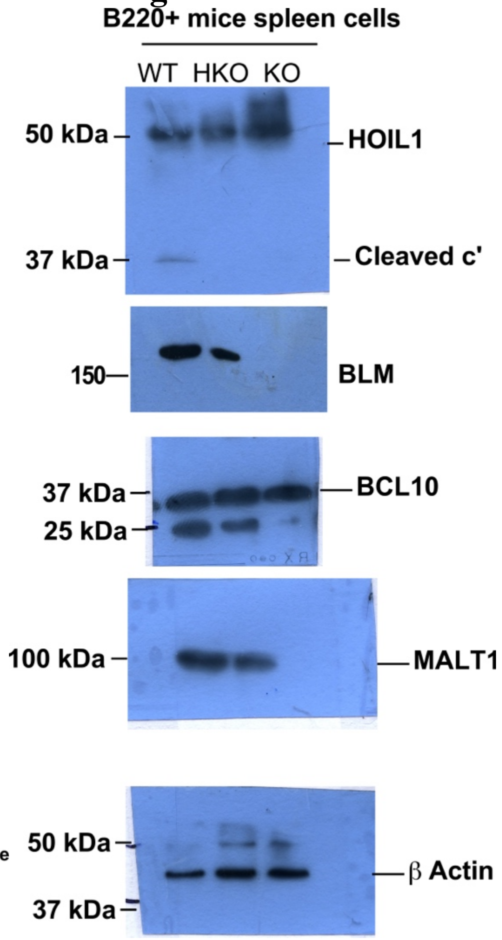

Figure 3I

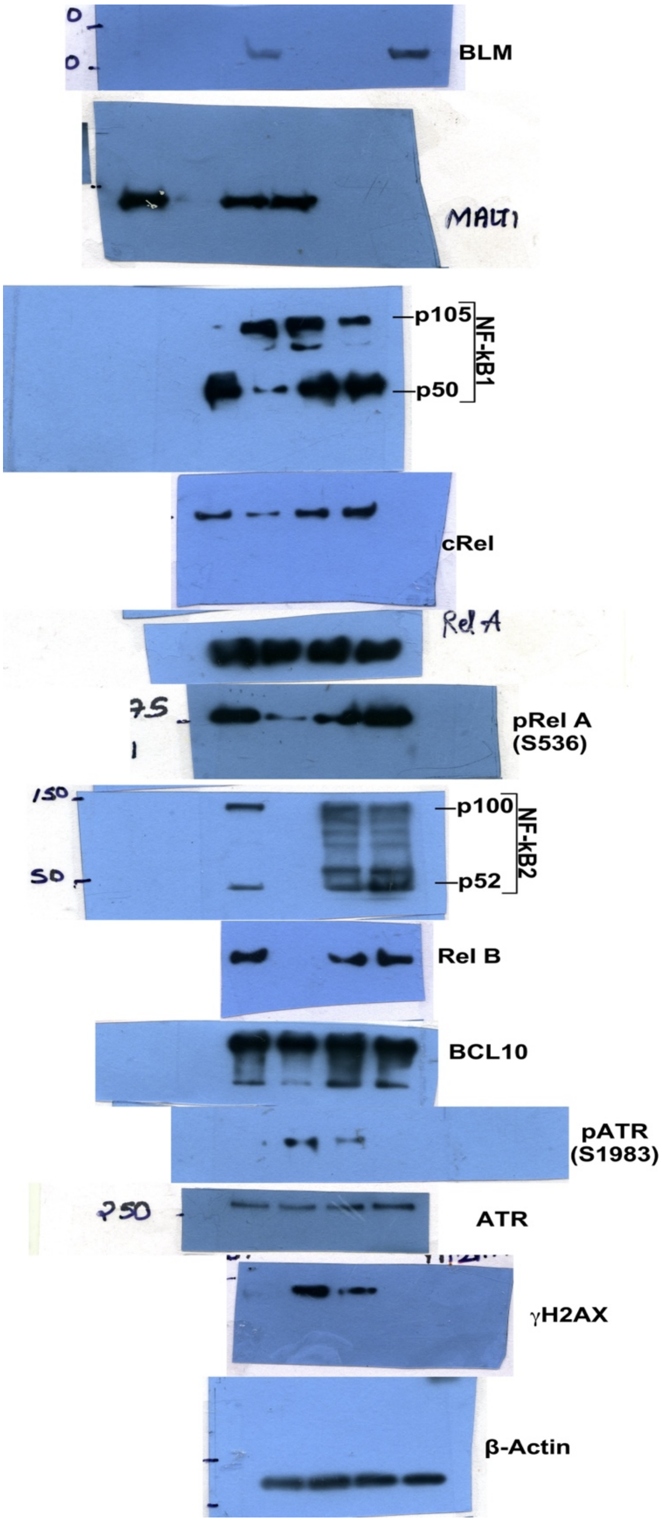

BLM
MALT1
p105
p50
NF-kB1
cRel
Rel A
75
pRel A (S536)
150
50
p100
p52
NF-kB2
Rel B
BCL10
pATR (S1983)
250
ATR
γH2AX
β-Actin

Figure 4G

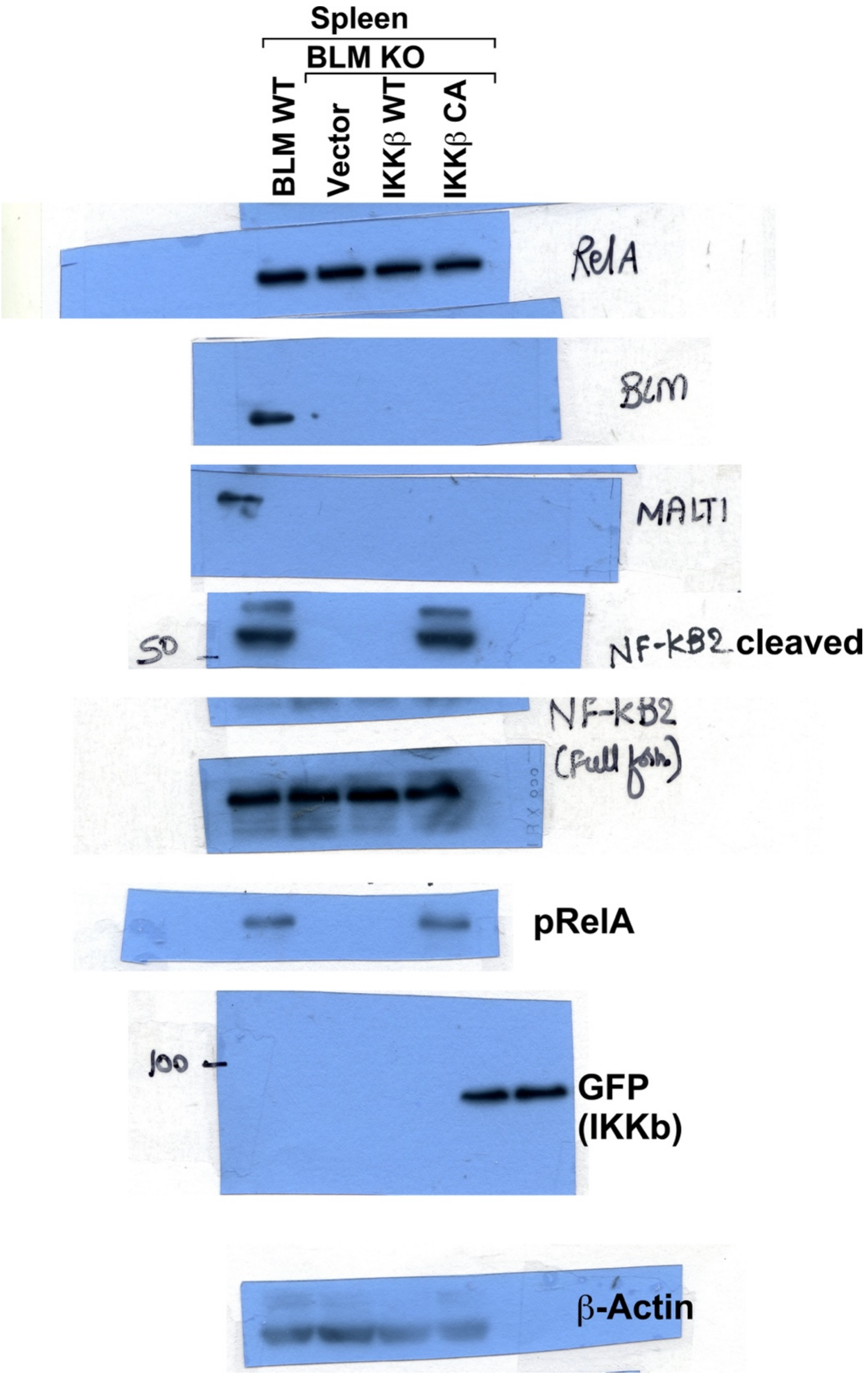

Spleen
BLM KO
BLM WT
Vector
IKKβ WT
IKKβ CA
RelA
BLM
MALT1
50
NF-KB2 cleaved
NF-KB2
(Full form)
pRelA
100
GFP
(IKKb)
β-Actin

Figure 5E

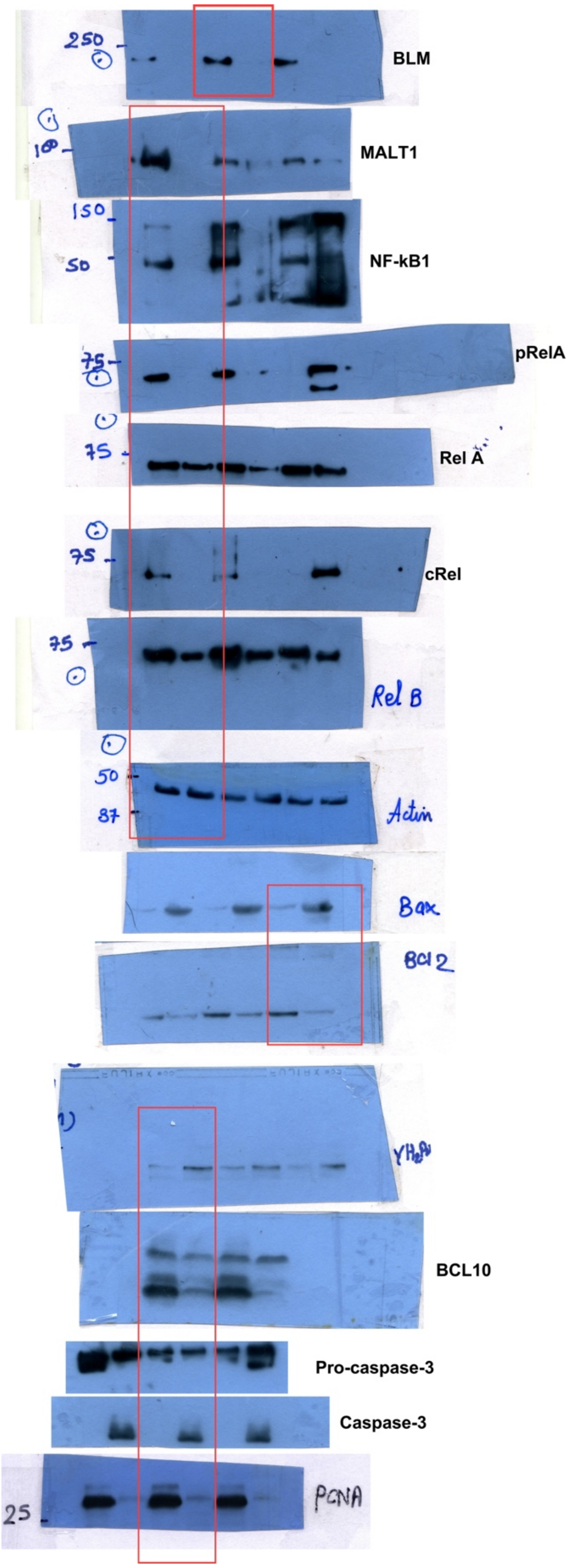

Figure 5J

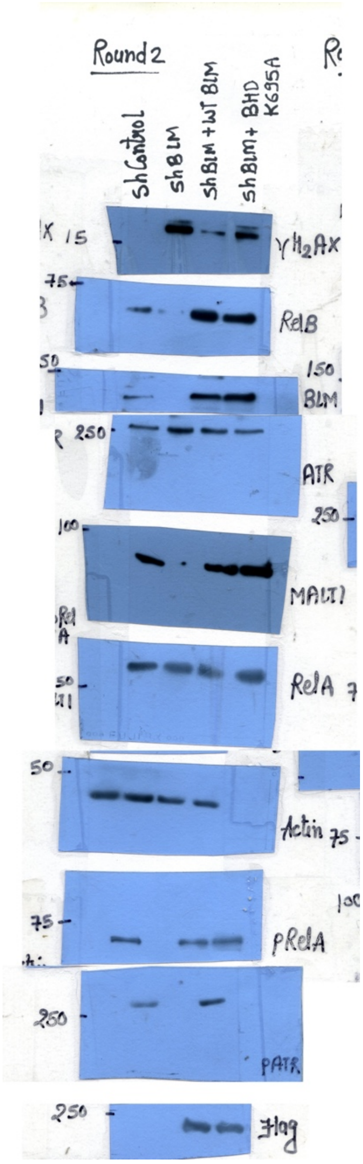

Figure 6I

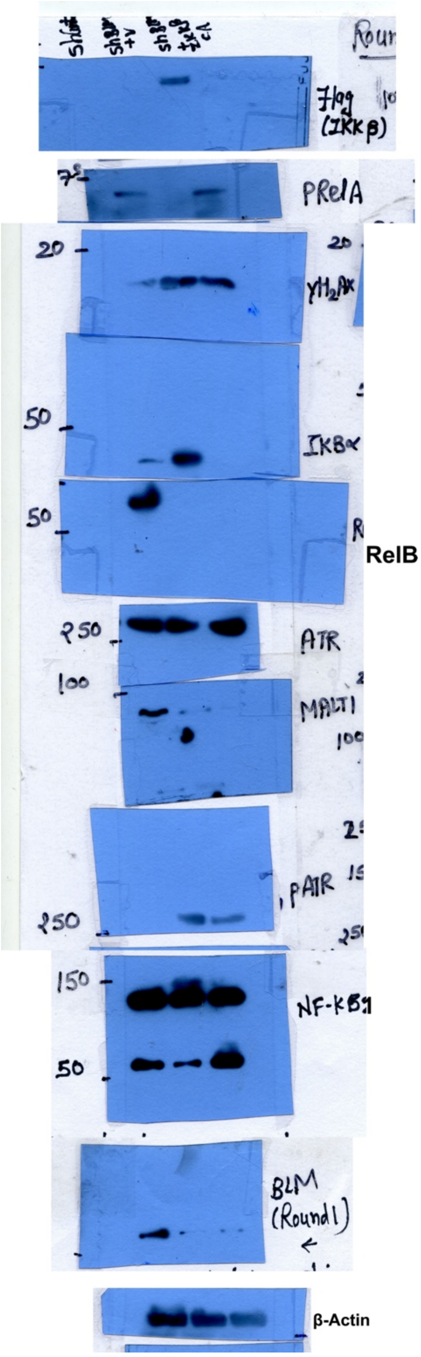
Flag
(IKKβ)
PRelA
20
γH2AX
50
IKBα
50
RelB
250
ATR
100
MALTI
250
pATR
150
NF-KB1
50
BLM
(Round1)
β-Actin

Figure S5C

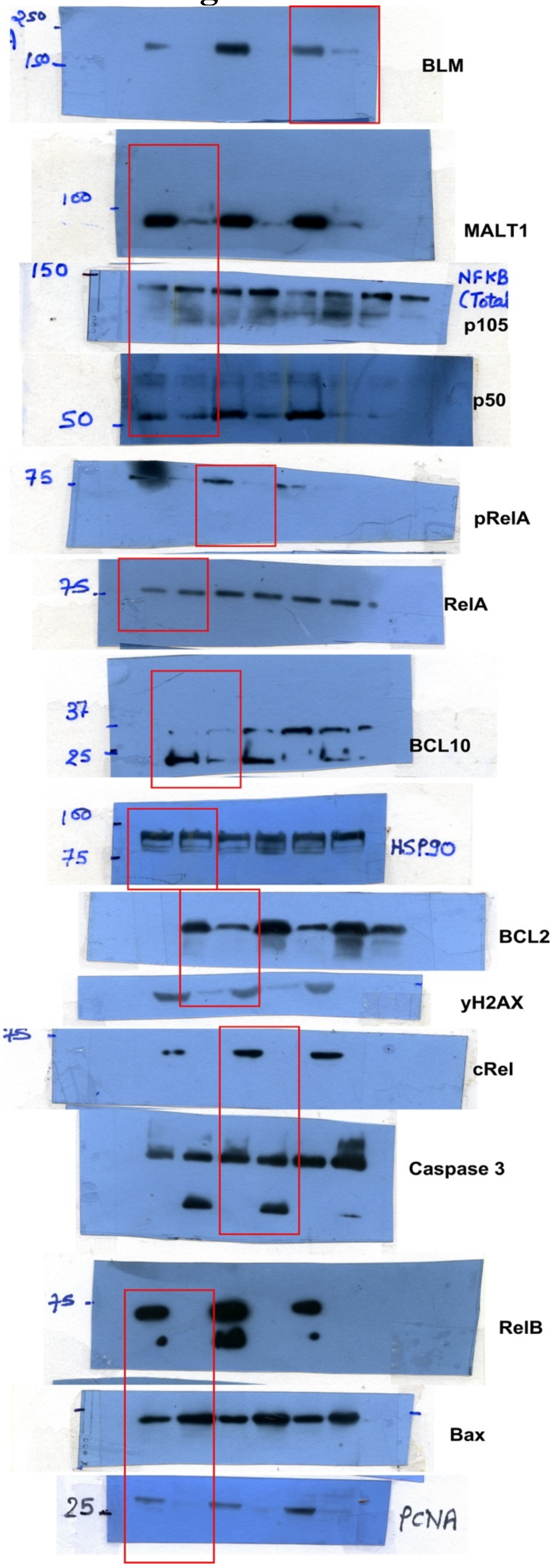
250
150
BLM
100
MALT1
150
NFKB
(Total
p105
p50
50
75
pRelA
75
RelA
37
25
BCL10
100
75
HSP90
BCL2
yH2AX
75
cRel
Caspase 3
75
RelB
Bax
25
PCNA

Figure S5G

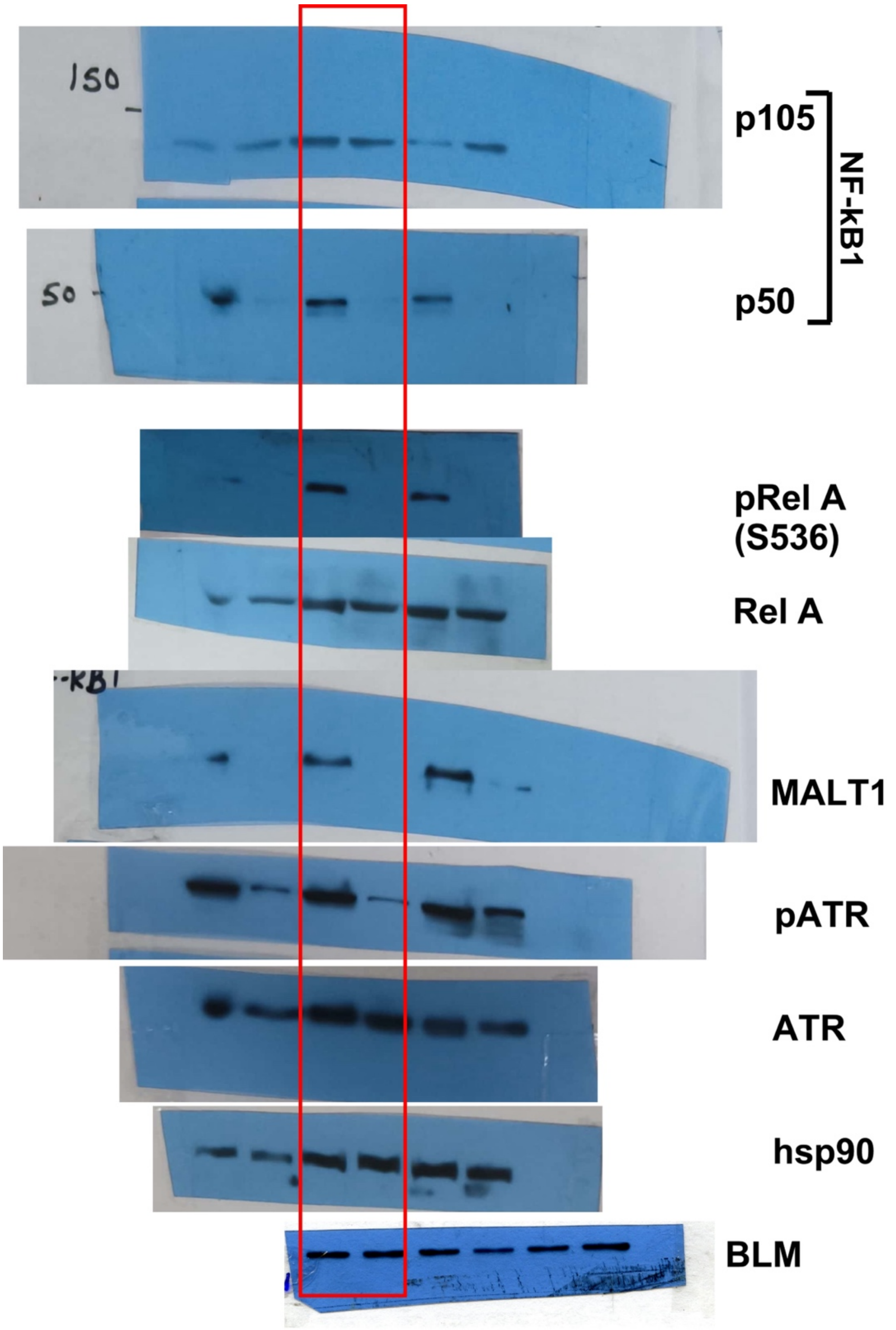
150
50
p105
p50
NF-kB1
pRel A
(S536)
Rel A
MALT1
pATR
ATR
hsp90
BLM

Figure S7A

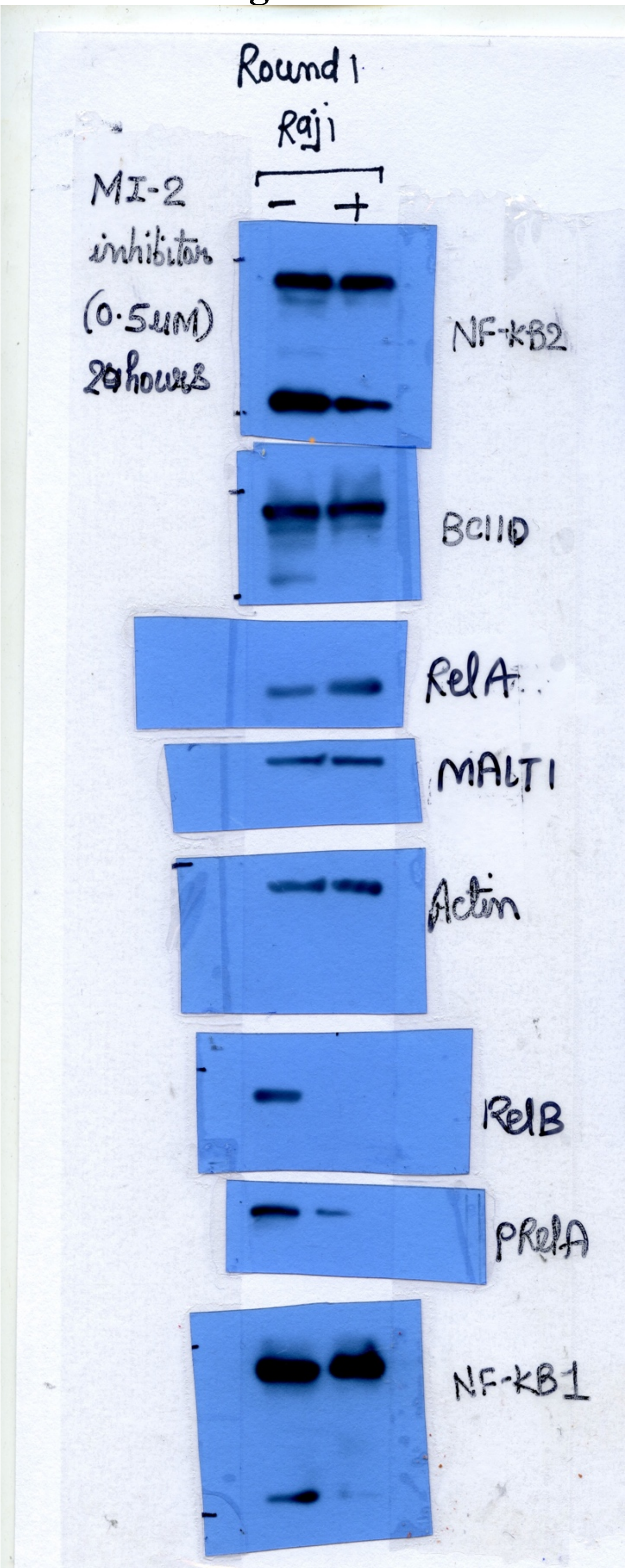

Round 1
Raji
MI-2 inhibitor (0.5uM) 20 hours
−
+
NF-κB2
Bcl10
RelA
MALT1
Actin
RelB
pRelA
NF-κB1
